# Characterization of Acetylation of Histone H3 at Lysine 9 in the Trigeminal Ganglion of a Rat Trigeminal Neuralgia Model

**DOI:** 10.1155/2022/1300387

**Published:** 2022-05-04

**Authors:** Wenbin Wei, Yuemin Liu, Yating Qiu, Minjie Chen, Yiwen Wang, Zixiang Han, Ying Chai

**Affiliations:** ^1^Department of Oral Surgery, Shanghai Ninth People's Hospital, Shanghai Jiao Tong University School of Medicine, Shanghai, China; ^2^College of Stomatology, Shanghai Jiao Tong University, Shanghai, China; ^3^National Center for Stomatology, Shanghai, China; ^4^National Clinical Research Center for Oral Diseases, Shanghai, China; ^5^Shanghai Key Laboratory of Stomatology, Shanghai, China

## Abstract

Trigeminal neuralgia (TN) is a chronic neuropathic pain disorder characterized by spontaneous and elicited paroxysms of electric-shock-like or stabbing pain in a region of the face. The epigenetic regulation of TN is still obscure. In current study, a rat TN model subject to carbamazepine (CBZ) treatment was established, and transcriptome- and genome-scale profiling of H3K9ac and HDAC3 was performed by RNA-seq and ChIP-seq. We observed that H3K9ac levels in the trigeminal ganglion were lower in the TN rats compared with those in the control, and CBZ treatment led to recovery of H3K9ac levels. Further, we found that HDAC3 was overactivated, which interfered with H3K9 acetylation due to higher phosphorylation in TN compared with that in the control. Finally, the phosphokinase leucine-rich repeat kinase 2 (LRRK2) was demonstrated to contribute to HDAC3 activity via the MAPK signaling pathway. Taken together, we identified a regulatory mechanism in which the phosphate groups transferred from activated ERK and LRRK2 to HDAC3 caused genome-scale deacetylation at H3K9 and resulted in the silencing of a large number of genes in TN. The kinases or important enzymes within this regulatory axis may represent important targets for TN therapy and prevention.

## 1. Introduction

Trigeminal neuralgia (TN) manifests as transient, paroxysmal but severe pain that occurs in the trigeminal nerve distribution area. TN is a common cause of facial pain in individuals aged over 50 years old. The incidence of TN in females is higher than in males. TN is frequently both misdiagnosed and underdiagnosed, and its incidence is variably reported between studies, with a range from 4.3 to 27 new cases per 100,000 people per year [1]. The compression of blood vessels at the trigeminal nerve has been observed in more than 30% of TN patients. The academic community has reached broad agreement that a neurovascular conflict with compression is the most common cause of classical TN [2]. In addition, the pathogenesis of TN involves neurodegeneration, epilepsy, and infection. A large number of neurophysiological, neuroimaging, and histological studies show that the underlying pathophysiological mechanism in TN involves the focal demyelination of primary trigeminal afferents near the entry of the trigeminal root into the pons [3, 4]. Such demyelination results in hyperexcitability of the focally demyelinated primary afferents due to the insufficiency of prompt resting potential re-establishment in axons.

An epigenetic trait is a stably heritable phenotype resulting from changes in a chromosome without alterations in the DNA sequence. Histone H3 is one of the most extensively modified of the five primary histone proteins, and acetylation at different lysine residues of histone H3 may play key roles in gene regulation. A previous study has reported larger numbers of acetylated H3K9-, H3K18-, and H3K27-positive cells in the TN group than in the sham-operation group [2, 5]. Interestingly, the notion that H3K9 global acetylation decreases in the Schwann cells in the trigeminal ganglia of a nerve injury mouse model induced by trigeminal inflammatory compression [6], whereas that histone deacetylase inhibitors (HDACi) can prevent the persistent hypersensitivity of neurons from neuropathic pain [7], which indicate that cells in different areas of trigeminal root entry zone and ganglion contain display a different response to TN. Moreover, various HDAC subclusters have also been found to exert varying effects on pain vulnerability and excitability of neurons [8, 9]. Therefore, the epigenetic regulation of the gene expression in response to compressive stress-induced imbalanced homeostasis in the trigeminal ganglion and trigeminal root entry zone is still obscure.

In the current study, we constructed a rat model of TN subject to carbamazepine (CBZ) treatment a widely used antiepileptic drug in clinical practice and performed transcriptome- and genome-scale H3K9ac profiling. CBZ as the first-line pharmacological treatment for trigeminal neuralgia [10] has been determined to function as HDACi [11]. Our study attempted to determine the alterative H3K9ac signature and elucidate the role of the *HDAC3* in TN [6, 7]. The large number of differentially expressed genes (DEGs) as well as the differential H3K9ac and *HDAC3* signals across the whole genome identified in this study reveals novel connections between H3K9ac and gene expression and the underlying mechanism of *HDAC3* in H3K9ac modulation, emphasizing the importance of epigenetic alterations in regulating transcription during TN.

## 2. Materials and Methods

### 2.1. Animal Study

Adult female Sprague-Dawley rats (200–220 g) were randomly divided into two groups: one group received chronic compression of the trigeminal nerve root only at left side (*n* = 30), and the other sham-operated group was considered as the negative control (*n* = 20). The TN model was prepared as previously described [12]. Five days after TN model establishment, the TN group was further divided into two groups: one was treated with 100 mg/kg/d CBZ (Sigma) dissolved into corn oil as previous described [13] for an additional five days (*n* = 15). All rats were sacrificed in the tenth day.

Orofacial mechanical allodynia testing was conducted at the same side of injured tergeminal nerve as previously described [5]. Rats were lightly restrained by their tail and allowed to stand on a mesh platform. The von Frey filaments (Ugo Basile) were applied to their left vibrissal pad border and cheek to collect baseline mechanical thresholds. Stimulation always began with the filament that produced the lowest force (bending forces of 0.008–6 g) and stopped when the threshold was found within the vibrissal pad of the rats. Each rat was tested five times at intervals of 3–5 s. The face grooming episodes (ipsilateral, contralateral, or bilateral face grooming) of the rats in the three groups were recorded by a video camera. Video recording of face grooming was started 20 minutes after the rats adapted to the test cage. The face grooming behavior was analyzed offline by an independent observer who was blind to the treatment administered to the rats. For each observation session of the rats over 10 minutes, the total duration of asymmetrical face grooming episodes as unilateral strokes with the dominant paw was counted [14]. For open field test, the animals were placed into an open-field hardboard box for 6 min individually to assess the motor activity, exploratory behavior, and emotional state. During the original period, 1 min was allowed for accommodation, and the next 5 min was for recording, including both horizontal and vertical movement and dwell time. In addition, the mice were allowed optional movement in the box during the experiment.

Rats were anesthetized with 60 mg/kg pentobarbital and shaved the hair and made a mid-line scalp incision with equal distances anterior and posterior to the center of the eyes to expose the skull and nasal bone. After dissecting the edge of the orbit free formed by the maxillary, frontal, lacrimal, and zygomatic bones, infraorbital nerve was dissected freely from the surrounding connective tissue for the consequent experiments. The animal study was reviewed and approved by the Medical Ethics Committee of Shanghai Jiao Tong University School of Medicine.

### 2.2. Immunofluorescence (IF) Assay

Frozen trigeminal nerves sections (10 *μ*m) were cut by a cryostat (Thermo Fisher Scientific). Unlike the nerve root region with the surrounding distribution of nuclei, the trigeminal ganglion contains robust nuclei in central region. Sections were fixed with 4% solution of paraformaldehyde and washed with phosphate-buffered saline (PBS) and then permeabilized with 0.1% Triton-X 100 (Millipore) and blocked with 5% horse serum in PBS. Immunostaining of samples was performed using antibodies against H3K9ac (1: 500, CST, #9649) or GFAP (1: 200, CST, #3670) overnight at 4°C. After washing with PBS for 4 times (5 min per time), the secondary goat antibodies against rabbit (for H3K9ac) or mouse (for GFAP) (1 : 20000, Jackson ImmunoResearch) were used, and incubation was performed for 2 h at room temperature; this was followed with further incubation with DAPI for 15 min and washing with PBS four times. After drying in air, mounting medium (Thermo Fisher Scientific) was added dropwise to the tissue slices, and they were coated with cover slips. The images were captured by fluorescence microscopy DM2000 (Leica Biosystems). Positive staining was statistically analyzed using ImageJ.

### 2.3. RNA Sequencing (RNA-seq)

Trigeminal ganglion tissues were stored in 1 mL TRIzol (Thermo Fisher Scientific) and ground in liquid nitrogen. Next, 100 *μ*L chloroform was added, and the cells were fully mixed and centrifuged at the highest speed at 4°C for 10 min. The supernatant was transferred into a new tube, isopropanol was added to the same volume, and cells were centrifuged at the highest speed at 4°C for 10 min. The precipitate was washed with 75% cold ethanol and dissolved in DEPC water. The concentration and quality of RNA were measured using a Nanodrop 2000 (Thermo Fisher Scientific) and an Agilent Bioanalyzer 2100 (Agilent). A total of 4 *μ*g of RNA from each group was used for library preparation using the NEBNext Ultra Directional RNA Library Prep Kit for Illumina (NEB) following the manufacturer's instructions and sequenced on an Illumina HiSeq platform.

The raw data were trimmed for adaptors, and low-quality reads were filtered out using Trimmomatic [15]. The quality of clean reads was checked using FastQC [16]. Next, clean reads were aligned to the latest rat genome assembly Rnor6.0 using Hisat2 [17]. The transcripts were assembled, and the expression levels were estimated with FPKM values using the StringTie algorithm with default parameters [18]. Differential mRNA and lncRNA expression among the groups were evaluated using the *R* package Ballgown [19], and the significance of differences was computed using the Benjamini and Hochberg (BH) *p* value adjustment method [20]. Gene annotation was performed using the Ensembl Genome Browser database (http://www.ensembl.org/index.html). The *R* package ClusterProfiler was used to annotate the DEGs using Gene Ontology (GO) terms and Kyoto Encyclopedia of Genes and Genomes (KEGG) pathways [21].

### 2.4. Chromatin Immunoprecipitation Sequencing (ChIP-Seq)

In brief, trigeminal ganglion tissues were incubated with 1 mL IP buffer (20 mM HEPES (pH 7.9), 350 mM NaCl, 0.1% NP-40, 1 mM DTT, 0.2 mM PMSF, 2 mg/mL leupeptin, and 2 mg/mL aprotinin) [22, 23] on ice for 30 min; then, the nuclei were harvested by centrifugation at the highest speed. The supernatant containing nuclei were sonicated to break up the genomic DNA into 200–500 bp fragments. Next, 10% lysates were saved as input, and the remaining were incubated with 1 *μ*g IP-grade antibodies of H3K9ac (CST, #9649) and *HDAC3* (Abclonal, A19537) at 4°C overnight. This was followed by incubation for 2 h with protein A beads at 37°C to pull down the bound DNA fragments.

For high-throughput sequencing, we added 3′-dA overhangs to the H3K9ac or *HDAC3* enriched or input DNA and ligated them to the adapter to build a DNA library. DNA libraries with ligated adapters were isolated based on the appropriate size for sequencing, using the Illumina Hiseq2000 platform. The raw sequence reads of input and IP were trimmed based on adaptors, and low-quality reads were filtered out using Cutadapt (v1.9.1) and Trimmomatic (v0.35). The quality of the clean reads was checked using Fastqc. The clean reads were mapped to the rat genome (assembly Rnor6.0) using the Bowtie 2 (v2.2.6) algorithm [24], and peak calling (*p* < 0.01) was performed using MACS 2 (v2.1.1) [25]. The differentially bound genes were analyzed based on *p* values less than 0.05 and annotated using DiffBind [26]. The relevant peaks on the genomic loci were visualized using the Integrative Genomics Viewer (IGV). GO analysis was used to determine the biological functions of genes associated with the differential peaks [27]. The raw ChIP sequencing data were submitted to the ArrayExpress database and registered under the accession number E-MTAB-10792 and 10793.

### 2.5. Immunoprecipitation (IP)

Previous steps were similar to those followed for the ChIP-seq assay. Trigeminal ganglia were harvested and mixed with 1 *μ*g phosphoserine/threonine (Abcam, ab17464), HDAC3 (CST, #85057), LRRK2 (CST, #13046), or IgG Rabbit IgG antibody and 40 *μ*l flurry IgA beads (Thermo Fisher Scientific) for rotating overnight at 4°C. Immunoprecipitates were washed with IP buffer (20 mM HEPES (pH 7.9), 350 mM NaCl, 0.1% NP-40, 1 mM DTT, 0.2 mM PMSF, 2 mg/mL leupeptin, and 2 mg/mL aprotinin) and purified with RIPA buffer (50 mM Tris (pH 7.4), 150 mM NaCl, 1% NP-40, 0.5% sodium deoxycholate, 0.1% SDS) with 1% proteasome inhibitor cocktail and 1% PMSF. The protein lysate was subjected to western blot assay.

### 2.6. Western Blot (WB) Assay

The protein lysate was then analyzed by SDS-PAGE and transferred to PVDF membranes (Bio-Rad Laboratories, Hercules, USA). The membrane was blocked with 5% fat-free milk in PBST for 30 min, followed by incubation overnight at 4°C with final dilutions of primary antibodies against p-HDAC1 (Ser421, Ser423) (1: 1000, Thermo Fisher Scientific, PA5-36911), HDAC1 (1: 2000, Abclonal, A19571), p-HDAC2 (Ser394) (1: 1000, Thermo Fisher Scientific, PA5-105021), HDAC2 (1: 2000, Abclonal, A19626), p-HDAC3 (Ser424) (1: 1000, Thermo Fisher Scientific, PA5-99339), HDAC3 (1: 2000, Abclonal, A19537), p-HDAC8 (Ser29) (1: 1000, Thermo Fisher Scientific, PA5-105031), HDAC8 (1: 2000, Abclonal, A5829), p-CK2*β* (Ser209) (1: 1000, Thermo Fisher Scientific, 44-1090G), CK2 (1: 2000, Abclonal, A2869), p-LRRK2 (Ser935) (1: 1000, Thermo Fisher Scientific, PA5-114599), LRRK2 (1: 2000, Abclonal, A17253), p-SRC (Tyr529) (1: 1000, Thermo Fisher Scientific, PA5-97356), SRC (1: 2000, Abclonal, A19119), p-ERK1/2 (Thr202, Tyr204)(1: 1000, Abclonal, AP1120), ERK1/2 (1: 2000, Abclonal, A19630), p-MEK (Ser271, Tyr275) (1: 1000, Abclonal, AP0535), MEK (1: 2000, Abclonal, A12950), or GAPDH (1: 2000, Proteintech Group, #60004-1). Next, the membrane was washed three times and then incubated with HRP-conjugated secondary antibodies (Proteintech Group). Membranes could be stripped using stripping buffer (Abcam, ab270550) at 52°C for 30 min via gently shaking, washed by PBST, then blocked by 5% fat-free milk for 2 h, and reincubated by antibodies at 4°C overnight. The blotting bands were developed with ECL plus immunoblotting detection reagents (Thermo Fisher Scientific) using UVP Chemstudio Plus System (Analytik Jena) and captured using ImageJ.

### 2.7. HDAC Activity Assay

*HDAC* activity/inhibition assay kits for *HDAC1* (Abx155637), *HDAC2* (Abx258089), *HDAC3* (Abx257885), and HDAC8 (Abx515264) were purchased from AmyJet Scientific Co. Ltd. Trigeminal ganglion tissues were incubated with RIPA buffer (50 mM Tris (pH 7.4), 150 mM NaCl, 1% NP-40, 0.5% sodium deoxycholate, 0.1% SDS) with 1% proteasome inhibitor cocktail and 1% PMSF. The protein concentration was quantified using the BSA method. The volume of different samples was adjusted to ensure that the total protein was identical. HDAC activities were processed following the manufacturer's instructions.

### 2.8. Statistical Analysis

Data are presented as the mean ± standard deviation for three independent experiments. The differences in values were analyzed using one-way ANOVA. Statistical significance was set at *p* value less than 0.05. All analysis is performed by SPSS 20.0.

## 3. Results

### 3.1. Decreased H3K9ac Profiling in Trigeminal Ganglia in TN Rats

**Figure 1:**
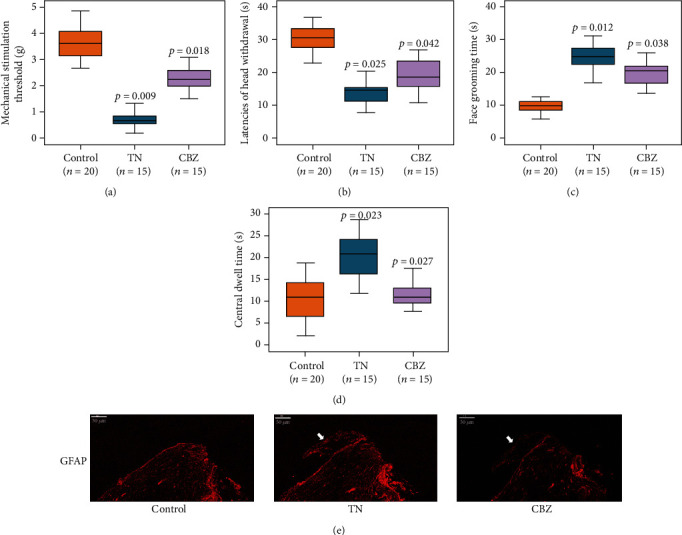
Confirmation of the TN rat model. (a)–(d) Behavior examination of orofacial mechanical allodynia by stimulation threshold and head withdrawal frequency and face grooming as well as open field test by central dwell time in the control (*n* = 20), TN rat model (*n* = 15), and TN rat model administered CBZ treatment (*n* = 15). The *p* values were given by the comparison between control and TN, as well as TN and CBZ. (e) GFAP staining in trigeminal root entry zone by IF (×200 magnification); white arrows indicate distal extension of GFAP-positive astrocytic process in the trigeminal root.

Thirty adult female SD rats were subjected to mechanical compression force of the trigeminal root entry zone by retrograde insertion of a plastic filament from the right inferior orbital fissure for the TN model. After five days, half of them were treated with 100 mg/kg/d CBZ for an additional five days. Behavioral examination of orofacial mechanical allodynia showed that, compared with the 20 rats in the control group, stimulus intensity (*F* = 6.681, *p* = 0.009; *F* = 4.412, *p* = 0.018) and mechanical hyperalgesia (*F* = 3.447, *p* = 0.025; *F* = 2.893, *p* = 0.042) were increased after the operation and attenuated at day 10 following CBZ treatment (Figures 1(a) and 1(b)). Likewise, the frequency of face-grooming behavior was also significantly increased after the operation and alleviated at day 10 with CBZ treatment (*F* = 6.244, *p* = 0.012; *F* = 3.055, *p* = 0.038) (Figure 1(c)). Open field test showed that CBZ could compromise the hyperactivity from TN effect (*F* = 3.628, *p* = 0.023; *F* = 3.314, *p* = 0.027) (Figure 1(d)). Nonetheless, we noticed that CBZ did not cause behavioral retardation in rats compared to control, indicating that the probable side effects of CBZ on rats' activity with the current dose did not exist in our system. Moreover, a distal extension of glial fibrillary acidic protein (GFAP) staining at the trigeminal root entry zone in the TN model at day 5 relative to the control indicated that astrocytes started to break through the boundary established by Schwann cells (Figure 1(e)). These results suggest the successful establishment of a rat TN model for subsequent experiments.

**Figure 2:**
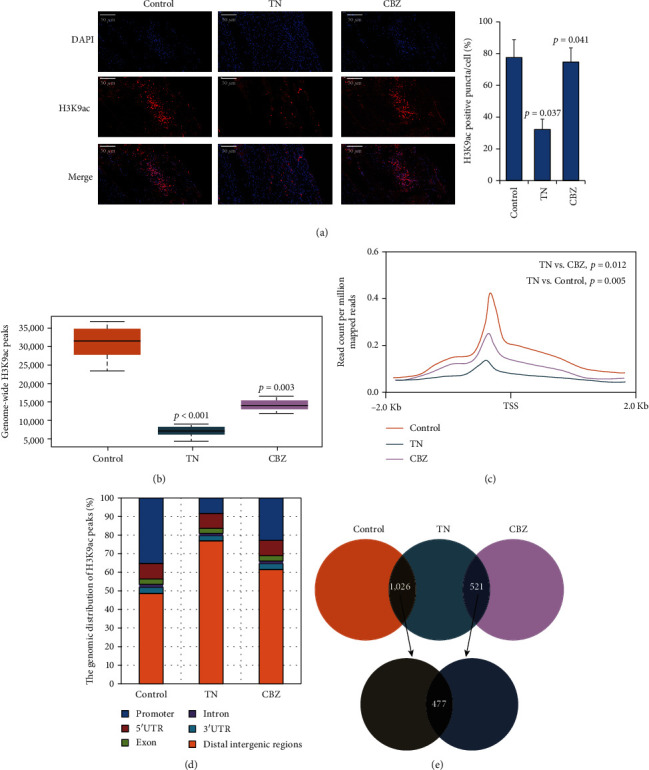
Overview of H3K9ac enrichment in the TN rat model. (a) Global H3K9ac staining in the trigeminal ganglion of the control, TN rat model, and TN rat model administered CBZ treatment (×200 magnification). The *p*-values were given by the comparison of H3K9ac positive puncta occupied in all cells between control and TN, as well as TN and CBZ. (b) Overall calling peaks of H3K9ac in the rat genome of control rats, TN rat model, and TN rat model administered CBZ treatment, by ChIP-seq. The *p* values were given by the comparison of genomic H3K9ac peak counts between control and TN, as well as TN and CBZ. (c) Metagene profiles of genome-wide H3K9ac in the control, TN rat model, and TN rat model administered CBZ treatment. (d) The distribution of differentially H3K9ac-enriched regions in the genomic contexts of the control, TN rat model, and TN rat model administered CBZ treatment. (e) Diagram showing the intersection of genes with differential H3K9ac enrichment in the control, TN rat model, and TN rat model administered CBZ treatment.

Next, H3K9ac was investigated by IHC and ChIP-seq. We observed fewer H3K9ac-positive nuclei within the trigeminal ganglia of the TN model than in the control group (*p* = 0.037) and in rats subjected to CBZ treatment (*p* = 0.041) (Figure 2(a)). Consistent with this result, genome-wide H3K9ac profiling also indicated weakened global H3K9ac peaks on neuron genomes in the TN group relative to the control (*p* < 0.001) and CBZ treatment groups (*p* = 0.003) (Figure 2(b)). Furthermore, H3K9ac density suggested that a unique subset of target genes exhibited compromised H3K9ac enrichment mainly at promoter regions (defined as −2 kb to +2 kb from the transcriptional start site [TSS]) in TN relative to the control (*p* = 0.005) and CBZ treatment groups (*p* = 0.012) (Figures 2(c) and 2(d)). Numerous H3K9ac-targeted sites identified by examining the differences between control or CBZ treatment and TN were mapped to 477 genes, with 421 genes showing a decrease and 56 an increase in H3K9ac in TN (fold change (FC) > 2 or <0.5, *p* < 0.05) (Figure 2(e)). Taken together, our findings suggested that TN causes reduced H3K9ac in the genomes of trigeminal ganglion neurons.

### 3.2. Transcriptome of Trigeminal Ganglion Affected by H3K9ac in TN

**Figure 3:**
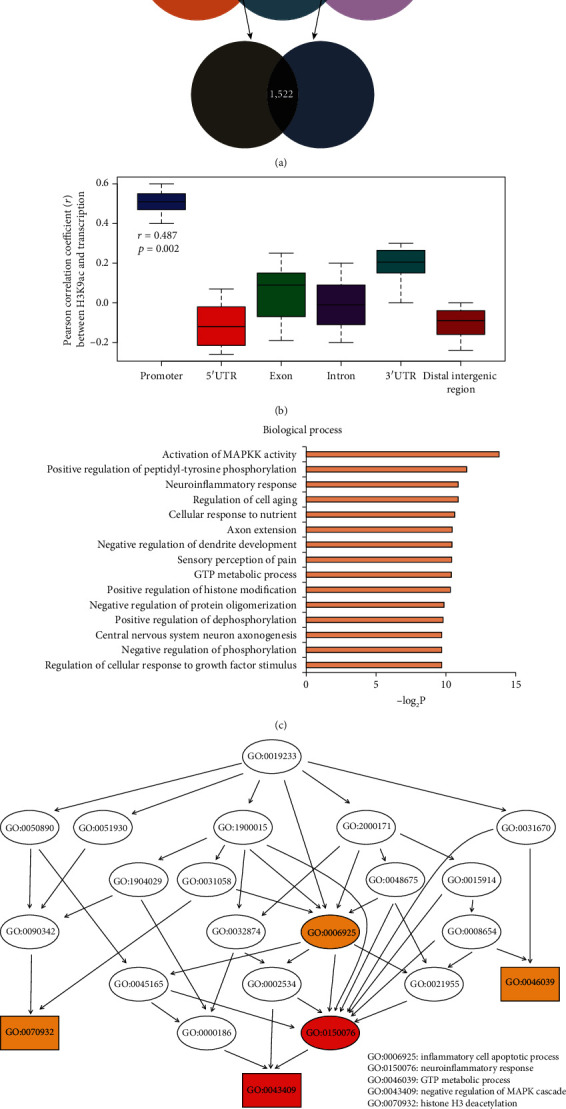
Overview of the transcriptome in the TN rat model. (a) Diagram showing intersecting DEGs in the control, TN rat model, and TN rat model administered CBZ treatment. (b) Correlation between RNA levels and H3K9ac enrichment at different genomic regions of DEGs, by Pearson correlation analysis. (c) Gene ontology analysis of DEGs with differential H3K9ac enrichment, for biological processes. (d) Regulatory connections between different terms of biological processes by directed acyclic graph. The key intermediate and terminal GO items are labeled by circle and box. *p* value less than 0.001 and 0.0001 are highlighted by orange and red.

To further study the expression of target genes affected by H3K9ac, we also conducted RNA-seq to investigate DEGs in TN. Based on the results, 164 genes showed higher expression while 1,358 genes had lower expression in TN relative to the control and CBZ treatment groups were characterized (FC > 2 or<0.5, *p* < 0.05) (Figure 3(a)). The genes with differential H3K9ac enrichment were all included among the DEGs. Pearson correlation analysis showed that only the differential H3K9ac enrichment at promoter regions correlated positively with the expression of the corresponding genes (*r* = 0.487, *p* = 0.002) (Figure 3(b)). Notably, GO analysis showed that these genes were tightly linked with functional regulation on axons or dendrites as well as inflammatory cytokine stimulus (Figure 3(c)). In particular, protein phosphorylated modification, phosphate metabolism process, and GTPase-associated activity occupied the central position in the directed acyclic graph of functional regulation (Figure 3(d)). Collectively, the data indicated that the abnormal H3K9ac contributed to the changes in the expression of a large proportion of genes in TN.

### 3.3. Excessive Activation of HDAC3 in Trigeminal Ganglia in TN

**Figure 4:**
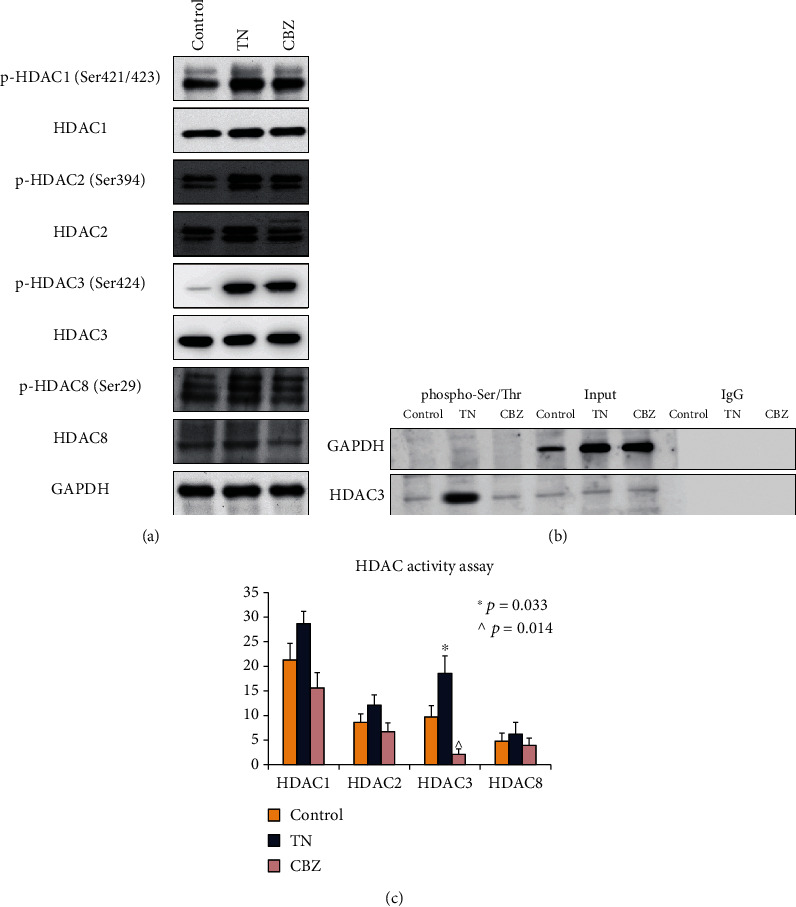
HDAC3 activity by phosphorylation modification in TN rat model. (a) Phosphorylation of HDAC1, 2, 3, and 8 in the control, TN rat model, and TN rat model administered CBZ treatment, by WB assay. (b) Phosphorylated HDAC3 profiles in the control, TN rat model, and TN rat model administered CBZ treatment, by phosphoserine and threonine IP assay. (c) The activity of HDAC1, 2, 3, and 8 in the control, TN rat model, and TN rat model administered CBZ treatment, by enzyme-linked immunosorbent assay (ELISA); “^∗^” and “^” represent comparison between TN and control as well as CBZ and TN with statistical significance.

Given that previous studies report seemingly controversial findings on histone H3 acetylation in neuralgia that the positive staining of H3K9, H3K18, and H3K27 acetylation were higher in postoperative days 7, 14, 21, and 28 in male TN rats [5], whereas the global acetylation of H3K9 decreased at day 21 by trigeminal inflammatory compression in mouse trigeminal ganglia [6], it is important to study the role of HDACs in regulating H3K9ac in TN. We focus on class I HDACs in the current study because of the evidence that class I HDACs (HDAC1, 2, 3, and 8) are widely implicated in H3K9ac transition in multiple human diseases and development [28–30]. The expression of class I HDACs was studied in TN by WB assay. Here, the expression of these four HDACs showed no significant difference between the control, TN, and CBZ treatment groups, and we unexpectedly found that the phosphorylation of *HDAC3* (Ser424) was higher in the TN and CBZ treatment groups than in the control (Figure 4(a)). Furthermore, the phosphorylated serine/threonine pull-down experiment showed that *HDAC3* was remarkably modified via phosphorylation in the TN and CBZ treatment groups relative to that in the control (Figure 4(b)). However, CBZ treatment strongly attenuated the activity of HDAC3 relative to that of HDAC1, 2, and 8 in the in vitro assay (Figure 4(c)). Unexpectedly, CBZ treatment produced a similar phosphorylation signature of *HDAC3* as in the TN group, despite substantially attenuating TN. We assumed that CBZ, being an HDACi, did not exert its effects by blocking phosphorylation.

**Figure 5:**
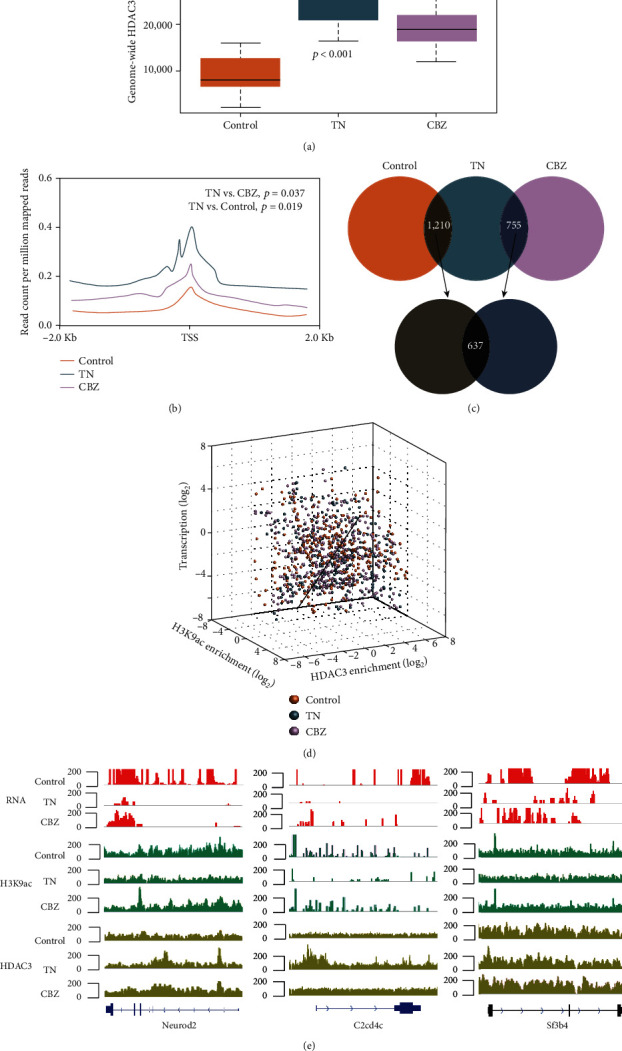
Overview of HDAC3 in TN rat model. (a) Overall calling peaks of *HDAC3* in the rat genome of the control, TN rat model, and TN rat model administered CBZ treatment, by ChIP-seq. (b) Metagene profiles of genome-wide *HDAC3* in the control, TN rat model, and TN rat model administered CBZ treatment. (c) Diagram showing intersecting genes with differential *HDAC3* enrichment in the control, TN rat model, and TN rat model administered CBZ treatment. (d) Correlation analysis of DEGs with differential H3K9ac and *HDAC3* enrichment in the control, TN rat model, and TN rat model administered CBZ treatment. (e) Gene-browser views of transcriptome, H3K9ac, and *HDAC3* profiles of *Neurod2, C2cd4c,* and *Sf3b4* in the control, TN rat model, and TN rat model administered CBZ treatment.

Next, we performed ChIP-seq analysis of trigeminal ganglia to investigate the genome-wide occupancy of *HDAC3* in TN. Opposite to the findings for enrichment of H3K9ac, global *HDAC3* occupancy was remarkably robust in TN compared with that in the control and CBZ treatment groups (Figure 5(a)). Our data indicate that the genomic binding ability of *HDAC3* was indeed strengthened, especially at the promoter regions in TN relative to the control (*p* = 0.019) and CBZ treatment groups (*p* = 0.037) (Figure 5(b)). Moreover, *HDAC3* peaks were mapped to 637 genes, in which 564 had increased, whereas 73 had decreased HDAC3 occupancy in TN compared to that in the control and CBZ treatment groups (FC > 2 or<0.5, *p* < 0.05) (Figure 5(c)). Hence, we identified three lists of genes with differentially occupied H3K9ac (477) and *HDAC3* (637) as well as DEGs (1, 522) by comparison between the TN and control group, and obtained an intersection of 288 genes. The transcription of these 288 genes was positively correlated with H3K9ac alteration (*r* = 0.506, *p* = 0.0017) and negatively correlated with *HDAC3* (*r* = −0.475, *p* = 0.0021) compared between control and TN groups, as well as positively correlated with H3K9ac alteration (*r* = 0.448, *p* = 0.0026) and negatively correlated with *HDAC3* (*r* = −0.626, *p* = 0.0013) compared between TN and CBZ groups (Figure 5(d)) (Table S1). Neurod2, C2cd4c, and Sf3b4, with the top correlation coefficient values, confirmed the enrichment of H3K9ac and *HDAC3* as well as their transcription using the IGV browser (Figure 5(e)). In conclusion, we found that the activated *HDAC3* contributed to the reduced H3K9ac modification and gene silencing in TN.

### 3.4. Phosphorylation of HDAC3 Regulated by LRRK2 via Compressive Stress-Induced MAPK/ERK Signaling Pathway

**Figure 6:**
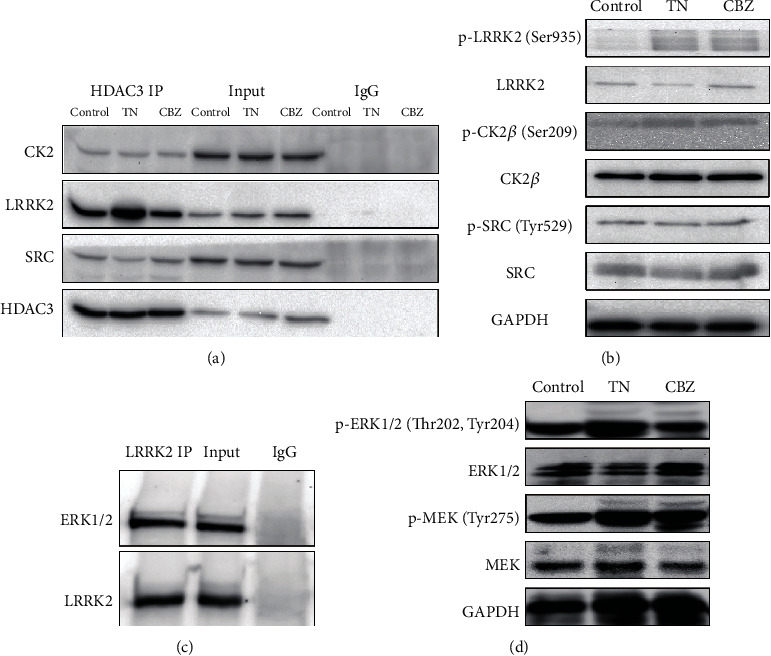
The activities of LRRK2 and MAPK signaling pathways in TN rat model. (a) The interaction between *LRRK2* and *HDAC3* in TN, by *HDAC3* IP assay. (b) Phosphorylation of *LRRK2*, *CK2β*, and *SRC* in the control, TN rat model, and TN rat model administered CBZ treatment, by WB assay. (c) Interaction between *LRRK2* and *ERK1/2* in TN, by *LRRK2* IP assay. (d) Phosphorylation of *MEK* and *ERK1/2* in the control, TN rat model, and TN rat model administered CBZ treatment, by WB assay.

As previously described, three enzymes, namely, casein kinase 2 (*CK2*) [31], leucine-rich repeat kinase 2 (*LRRK2*) [32], and SRC protooncogene, nonreceptor tyrosine-protein kinase (SRC) [33], contributing to the phosphorylation of *HDAC3* or *HDAC3-H1.3* complex were investigated to elucidate the mechanism underlying the phosphorylation of *HDAC3* in TN. Initially, we determined a substantial interaction between *HDAC3* and *LRRK2* rather than *CK2* or *SRC*, by IP study. In addition, the binding ability of *LRRK2* with *HDAC3* strengthened in TN and under CBZ treatment compared with that in the control (Figure 6(a)). Next, the protein expression of these kinases did not change between the control, TN, or CBZ treatment groups, but robust phosphorylation of *LRRK2* (Ser935) was observed in TN and CBZ treatment groups relative to the control (Figure 6(b)). To trace back to the phosphate groups from *LRRK2*, *LRRK2* pull-down and IP assays of trigeminal root entry zone of TN were conducted to detect a variety of enzymes that catalyze phosphate group transmission. We noticed that *MAPK/ERK* signaling pathway was the top affected item in TN (Figure 3(c)) and was reported to participate in pathogenesis neuralgia in previous studies [34, 35]. We harvested *ERK1/2* from the *LRRK2* binding proteins (Figure 6(c)). Consistent with this, the high expression of phosphorylated *MEK* and *ERK1/2* indicated that the *MAPK/ERK* signaling pathway was indeed activated in TN and CBZ treatment groups compared with that in the control (Figure 6(d)).

## 4. Discussion

A large number of preclinical and clinical studies show that histone acetyltransferases (HATs) and HDACs play an important role in the development of neuralgia [36]. HDACs have a significant analgesic effect, thus drawing much attention from researchers. *HDAC1* levels increase in the spinal cord of the spinal nerve ligation rat model, where *HDAC1* interacts with a heterodimer composed of c-Jun, finally activating the *JNK* signaling pathway to participate in the maintenance of neuralgia. LG325 (*HDAC1* inhibitor) can suppress the activity of c-Jun to alleviate pain [37]. Furthermore, D-*β*-hydroxybutyric acid is reported to not only relieve mechanical and thermal hyperalgesia in rats but also to improve their motor function by reversing the presence of low acetylation and expression of *FOXO3a*, catalase, and *SOD2* in the damaged area [38]. Substantial attenuating effects of CBZ on TN are observed in this study. The number of DEGs in our RNA-seq data, consistent with that reported in a previous study [39], is far higher than genes with differential H3K9ac occupancy, indicating that in addition to H3K9ac, the acetylation levels of other histones also decline in TN.

Besides the well-acknowledged inflammatory response and neuronal self-repair in TN [40], the biological functions associated with *MAPK* activity and the GTP metabolic process are highlighted in our sequencing data, prompting us to study the activity of HDACs from phosphorylation modification. We only observe obvious phosphorylation at Ser29 of *HDAC3* in this system. The IP experiments showed one clear band of HDAC3 within the phosphor-Ser/Thr pull down fraction. However, *HDAC3* in general has multiple serine and threonine phosphorylation sites [33]. Therefore, we suspect that the activation of *HDAC3* by phosphorylation at different sites determines individual and specific functions in diseases and other biological events.

Consistent with sequencing data, the findings show that the *MAPK/ERK* signaling pathway substantially contributes to providing phosphate groups to *LRRK2* and *HDAC3*. Here, we raise the interesting topic of kinase selection for *HDAC3* phosphorylation in TN. C*K2, LRRK2*, and *SRC* are the phosphokinases for other HDACs, and they govern the phosphorylation of *HDAC3* in different tissues [41]. It is challenging to elucidate the mechanism underlying such behavior under special conditions. Furthermore, *CK2* and *SRC* are likely to play a role in other HDACs in the pathogenesis of TN.

**Figure 7:**
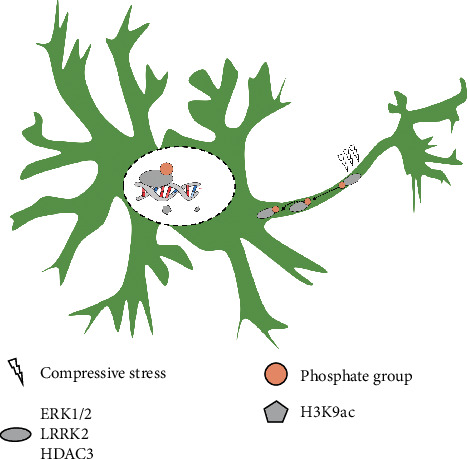
The graphic overview of this study. The phosphate groups transported from activated MAPK/ERK pathway and LRRK2 to HDAC3 causes the repression of H3K9ac and results in a large number of gene expression silencing in trigeminal ganglion induced by compressive stress.

Overall, we identified a TN regulatory mechanism in which the phosphate groups transferred from activated *ERK* and *LRRK2* to *HDAC3* caused genome-scale deacetylation at H3K9 and resulted in the silencing of a large number of genes (Figure 7). The kinases or important enzymes within this regulatory axis represent potential targets for TN therapy and prevention.

## Data Availability

The datasets used and/or analyzed during the current study are available from the corresponding authors upon reasonable request.

## Supplementary Materials

Supplementary Materials Table S1: the list of DEGs with significant H3K9ac and HDAC3 enrichments. Candidate genes meet with the following conditions, FPKM values significantly differential (FC > 2 or<0.5, *p* < 0.05) comparison between control and TN groups as well as TN and CBZ groups in RNA-seq and ChIP-seq of H3K9ac and HDAC3.
