# Supplementary material for: Characterization of Acetylation of Histone H3 at Lysine 9 in the Trigeminal Ganglion of a Rat Trigeminal Neuralgia Model
Source: Oxid Med Cell Longev. 2022 May 4;2022:1300387. doi: 10.1155/2022/1300387 (PMC9095355; doi:10.1155/2022/1300387)
Supplement: Supplementary Materials — Table S1: the list of DEGs with significant H3K9ac and HDAC3 enrichments. Candidate genes meet with the following conditions, FPKM values significantly differential (FC > 2 or<0.5, p < 0.05) comparison between control and TN groups as well as TN and CBZ groups in RNA-seq and ChIP-seq of H3K9ac and HDAC3. [file 1300387.f1.pdf]

Table S1. The list of DEGs with significant H3K9ac and HDAC3 enrichments.

GeneName	Annotation
AL731706.1	novel protein
Sspo	SCO-spondin
Gm45785	predicted gene 45785
Mettl7b	methyltransferase like 7B
Fgf21	fibroblast growth factor 21
Npcd	neuronal pentraxin chromo domain
Neurod2	neuronal differentiation 2
Trem3	triggering receptor expressed on myeloid cells 3
Kif21b	kinesin family member 21B
Cass4	Cas scaffolding protein family member 4
Gm44973	predicted gene 44973
Rec114	REC114 meiotic recombination protein
Adm2	adrenomedullin 2
Olfr996	olfactory receptor 996
Igf2bp3	insulin-like growth factor 2 mRNA binding protein 3
C2cd4c	C2 calcium-dependent domain containing 4C
G0s2	G0/G1 switch gene 2
Rad21l	RAD21-like (S. pombe)
Rdh9	retinol dehydrogenase 9
Cdk15	cyclin-dependent kinase 15
Nyx	nyctalopin
P2rx7	purinergic receptor P2X, ligand-gated ion channel, 7
Bdkrb2	bradykinin receptor, beta 2
Calcb	calcitonin-related polypeptide, beta
Gm13539	predicted gene 13539
Gm10378	predicted gene 10378
Vmn1r34	vomeronasal 1 receptor 34
Vmn1r32	vomeronasal 1 receptor 32
Aknad1	AKNA domain containing 1
Fut1	fucosyltransferase 1
Entpd2	ectonucleoside triphosphate diphosphohydrolase 2
Lcp2	lymphocyte cytosolic protein 2
Mef2b	myocyte enhancer factor 2B
Gpnmb	glycoprotein (transmembrane) nmb
Nnmt	nicotinamide N-methyltransferase
Calcrl	calcitonin receptor-like
Sf3b4	splicing factor 3b, subunit 4
Adgrd1	adhesion G protein-coupled receptor D1
Elfn2	leucine rich repeat and fibronectin type III, extracellular 2
1810010D01Rik	RIKEN cDNA 1810010D01 gene
Slc19a3	solute carrier family 19, member 3
Tyrobp	TYRO protein tyrosine kinase binding protein
Trib3	tribbles pseudokinase 3
Mcidas	multiciliate differentiation and DNA synthesis associated cell cycle protein
Dcn	decorin
Bex4	brain expressed X-linked 4
Gm9936	predicted gene 9936
Chst4	carbohydrate sulfotransferase 4
Tac4	tachykinin 4
Bglap3	bone gamma-carboxyglutamate protein 3

Arap3	ArfGAP with RhoGAP domain, ankyrin repeat and PH domain 3
Pvrig	poliovirus receptor related immunoglobulin domain containing
4930583I09Rik	RIKEN cDNA 4930583I09 gene
Nmrk2	nicotinamide riboside kinase 2
Mfsd4b1	major facilitator superfamily domain containing 4B1
Ephx4	epoxide hydrolase 4
Hpd	4-hydroxyphenylpyruvic acid dioxygenase
Gm21985	predicted gene 21985
Crp	C-reactive protein, pentraxin-related
Gm266	predicted gene 266
Gm21983	predicted gene 21983
Dennd2d	DENN/MADD domain containing 2D
Pcsk9	proprotein convertase subtilisin/kexin type 9
Fgfrl1	fibroblast growth factor receptor-like 1
Pla2g2e	phospholipase A2, group IIE
Cfap52	cilia and flagella associated protein 52
Gm49711	predicted gene, 49711
Aqp8	aquaporin 8
Cyp2b13	cytochrome P450, family 2, subfamily b, polypeptide 13
Gprc5d	G protein-coupled receptor, family C, group 5, member D
Lrrc74b	leucine rich repeat containing 74B
Pdzd7	PDZ domain containing 7
Kctd16	potassium channel tetramerisation domain containing 16
Aloxe3	arachidonate lipoxygenase 3
Vmn1r33	vomeronasal 1 receptor 33
1700001J03Rik	RIKEN cDNA 1700001J03 gene
Taf7l	TATA-box binding protein associated factor 7 like
Inhbe	inhibin beta-E
4930524B15Rik	RIKEN cDNA 4930524B15 gene
Zfp185	zinc finger protein 185
Mkrn2os	makorin, ring finger protein 2, opposite strand
Cth	cystathionase (cystathionine gamma-lyase)
Gpr160	G protein-coupled receptor 160
Mfsd4b3	major facilitator superfamily domain containing 4B3
Gm19345	predicted gene, 19345
Ces5a	carboxylesterase 5A
BC048671	cDNA sequence BC048671
Lcp1	lymphocyte cytosolic protein 1
1700123K08Rik	RIKEN cDNA 1700123K08 gene
Il1rn	interleukin 1 receptor antagonist
Ceacam16	carcinoembryonic antigen-related cell adhesion molecule 16
Spaca9	sperm acrosome associated 9
A730049H05Rik	RIKEN cDNA A730049H05 gene
Cmah	cytidine monophospho-N-acetylneuraminic acid hydroxylase
Sigirr	single immunoglobulin and toll-interleukin 1 receptor (TIR) domain
Hvcn1	hydrogen voltage-gated channel 1
Hrk	harakiri, BCL2 interacting protein (contains only BH3 domain)
Plag1	pleiomorphic adenoma gene 1
Pnma5	paraneoplastic antigen family 5
Tmc3	transmembrane channel-like gene family 3
Cox6a2	cytochrome c oxidase subunit 6A2
Stc2	stanniocalcin 2

Ncf4	neutrophil cytosolic factor 4
Irx1	Iroquois homeobox 1
Wt1	Wilms tumor 1 homolog
Fam129a	family with sequence similarity 129, member A
Steap1	six transmembrane epithelial antigen of the prostate 1
Gbx1	gastrulation brain homeobox 1
Apobec1	apolipoprotein B mRNA editing enzyme, catalytic polypeptide 1
Ddit3	DNA-damage inducible transcript 3
Ostn	osteocrin
Cfap58	cilia and flagella associated protein 58
Itgb2	integrin beta 2
Ceacam3	carcinoembryonic antigen-related cell adhesion molecule 3
1700016K19Rik	RIKEN cDNA 1700016K19 gene
Gm43738	predicted gene 43738
Odf3l1	outer dense fiber of sperm tails 3-like 1
Th	tyrosine hydroxylase
Gpr65	G-protein coupled receptor 65
Olfr692	olfactory receptor 692
Atf3	activating transcription factor 3
Atp2a3	ATPase, Ca++ transporting, ubiquitous
Cd27	CD27 antigen
Creb5	cAMP responsive element binding protein 5
Slc7a11	solute carrier family 7 (cationic amino acid transporter, y+ system), member 11
Ptpn18	protein tyrosine phosphatase, non-receptor type 18
Scd4	stearoyl-coenzyme A desaturase 4
Ascl3	achaete-scute family bHLH transcription factor 3
Angptl6	angiopoietin-like 6
Bpifb6	BPI fold containing family B, member 6
Nupr1	nuclear protein transcription regulator 1
Rab3il1	RAB3A interacting protein (rabin3)-like 1
Olfr1507	olfactory receptor 1507
Lrrc10	leucine rich repeat containing 10
Itk	IL2 inducible T cell kinase
Rorc	RAR-related orphan receptor gamma
Gfral	GDNF family receptor alpha like
Ascl2	achaete-scute family bHLH transcription factor 2
Pheta2	PH domain containing endocytic trafficking adaptor 2
Slc6a9	solute carrier family 6 (neurotransmitter transporter, glycine), member 9
Il2rb	interleukin 2 receptor, beta chain
Reep6	receptor accessory protein 6
Ugt3a2	UDP glycosyltransferases 3 family, polypeptide A2
Foxb2	forkhead box B2
Samsn1	SAM domain, SH3 domain and nuclear localization signals, 1
Ncmap	noncompact myelin associated protein
Aph1c	aph1 homolog C, gamma secretase subunit
Fibcd1	fibrinogen C domain containing 1
Klf15	Kruppel-like factor 15
Scel	sciellin
Zfp13	zinc finger protein 13
Bhmt2	betaine-homocysteine methyltransferase 2
Odf3	outer dense fiber of sperm tails 3
Gm648	predicted gene 648

Fam84b	family with sequence similarity 84, member B
Rora	RAR-related orphan receptor alpha
Fasl	Fas ligand (TNF superfamily, member 6)
Gpr132	G protein-coupled receptor 132
Olfr1349	olfactory receptor 1349
Pnpla3	patatin-like phospholipase domain containing 3
Htr3b	5-hydroxytryptamine (serotonin) receptor 3B
Mvd	mevalonate (diphospho) decarboxylase
Gch1	GTP cyclohydrolase 1
Btc	betacellulin, epidermal growth factor family member
Aqp9	aquaporin 9
Drp2	dystrophin related protein 2
Nos2	nitric oxide synthase 2, inducible
Fa2h	fatty acid 2-hydroxylase
Vcam1	vascular cell adhesion molecule 1
Gypa	glycophorin A
Csf2ra	colony stimulating factor 2 receptor, alpha, low-affinity (granulocyte-macrophage)
Aqp7	aquaporin 7
Tnk1	tyrosine kinase, non-receptor, 1
Olah	oleoyl-ACP hydrolase
Traf3ip3	TRAF3 interacting protein 3
Cdsn	corneodesmosin
Ptpn14	protein tyrosine phosphatase, non-receptor type 14
Celsr1	cadherin, EGF LAG seven-pass G-type receptor 1
Rhod	ras homolog family member D
Apol10b	apolipoprotein L 10B
Slc52a3	solute carrier protein family 52, member 3
4833423E24Rik	RIKEN cDNA 4833423E24 gene
AA467197	expressed sequence AA467197
Foxr1	forkhead box R1
Krt78	keratin 78
Hepacam	hepatocyte cell adhesion molecule
Pi16	peptidase inhibitor 16
Slc1a7	solute carrier family 1 (glutamate transporter), member 7
Dtna	dystrobrevin alpha
Fgb	fibrinogen beta chain
Grid2ip	glutamate receptor, ionotropic, delta 2 (Grid2) interacting protein 1
Zmynd10	zinc finger, MYND domain containing 10
Zcchc24	zinc finger, CCHC domain containing 24
Acox2	acyl-Coenzyme A oxidase 2, branched chain
Pdgfb	platelet derived growth factor, B polypeptide
Gm15319	predicted gene 15319
Dlg2	discs large MAGUK scaffold protein 2
Calca	calcitonin/calcitonin-related polypeptide, alpha
Fam50b	family with sequence similarity 50, member B
Entpd3	ectonucleoside triphosphate diphosphohydrolase 3
Eln	elastin
Sowahb	sosondowah ankyrin repeat domain family member B
Gif	gastric intrinsic factor
Fn3k	fructosamine 3 kinase
Plch2	phospholipase C, eta 2
Fam107a	family with sequence similarity 107, member A

Isg20	interferon-stimulated protein
Insrr	insulin receptor-related receptor
Rspo1	R-spondin 1
Sh2d3c	SH2 domain containing 3C
Dhh	desert hedgehog
Gm28035	predicted gene, 28035
Mettl27	methyltransferase like 27
Hmx3	H6 homeobox 3
Nrap	nebulin-related anchoring protein
Muc20	mucin 20
6030468B19Rik	RIKEN cDNA 6030468B19 gene
Gpr171	G protein-coupled receptor 171
Cpt1b	carnitine palmitoyltransferase 1b, muscle
Pkd1l1	polycystic kidney disease 1 like 1
Rdh16	retinol dehydrogenase 16
Fibin	fin bud initiation factor homolog (zebrafish)
Cracr2a	calcium release activated channel regulator 2A
Aldob	aldolase B, fructose-bisphosphate
Vipr2	vasoactive intestinal peptide receptor 2
Hgf	hepatocyte growth factor
Trim66	tripartite motif-containing 66
Mib2	mindbomb E3 ubiquitin protein ligase 2
Pkdrej	polycystin (PKD) family receptor for egg jelly
Gucy2e	guanylate cyclase 2e
Vrtn	vertebrae development associated
Maff	v-maf musculoaponeurotic fibrosarcoma oncogene family, protein F (avian)
Ifi44	interferon-induced protein 44
Enpp6	ectonucleotide pyrophosphatase/phosphodiesterase 6
Rgs8	regulator of G-protein signaling 8
Gabrr2	gamma-aminobutyric acid (GABA) C receptor, subunit rho 2
Rsph4a	radial spoke head 4 homolog A (Chlamydomonas)
Sdk2	sidekick cell adhesion molecule 2
Dll4	delta like canonical Notch ligand 4
Adtrp	androgen dependent TFPI regulating protein
Hmgcs1	3-hydroxy-3-methylglutaryl-Coenzyme A synthase 1
Lipc	lipase, hepatic
Tuba3a	tubulin, alpha 3A
N4bp2l1	NEDD4 binding protein 2-like 1
Sh3tc1	SH3 domain and tetratricopeptide repeats 1
Prlr	prolactin receptor
Lss	lanosterol synthase
Tnfrsf13c	tumor necrosis factor receptor superfamily, member 13c
Trpv1	transient receptor potential cation channel, subfamily V, member 1
Gm10439	predicted gene 10439
Olfr330	olfactory receptor 330
Fgfr4	fibroblast growth factor receptor 4
Zfp773	zinc finger protein 773
Ifnlr1	interferon lambda receptor 1
Gypc	glycophorin C
Rbpjl	recombination signal binding protein for immunoglobulin kappa J region-like
Eno2	enolase 2, gamma neuronal
Syt2	synaptotagmin II

Atp4a	ATPase, H+/K+ exchanging, gastric, alpha polypeptide
Styxl1	serine/threonine/tyrosine interacting-like 1
Ppargc1a	peroxisome proliferative activated receptor, gamma, coactivator 1 alpha
Sftpb	surfactant associated protein B
Samd4	sterile alpha motif domain containing 4
Ciart	circadian associated repressor of transcription
Nectin4	nectin cell adhesion molecule 4
Ntrk3	neurotrophic tyrosine kinase, receptor, type 3
Drc1	dynein regulatory complex subunit 1
Gad1	glutamate decarboxylase 1
Cplx3	complexin 3
Ppp1r15a	protein phosphatase 1, regulatory subunit 15A
Rnf43	ring finger protein 43
Grhl2	grainyhead like transcription factor 2
Vsig1	V-set and immunoglobulin domain containing 1
Olfr5	olfactory receptor 5
Soat2	sterol O-acyltransferase 2
Chac1	ChaC, cation transport regulator 1
Cd164l2	CD164 sialomucin-like 2
Gramd2	GRAM domain containing 2
Apobr	apolipoprotein B receptor
Tnfrsf22	tumor necrosis factor receptor superfamily, member 22
F3	coagulation factor III
Slco1b2	solute carrier organic anion transporter family, member 1b2
Idi1	isopentenyl-diphosphate delta isomerase
Olfr1508	olfactory receptor 1508
Hhipl1	hedgehog interacting protein-like 1
Atp6v1b1	ATPase, H+ transporting, lysosomal V1 subunit B1
Ldlr	low density lipoprotein receptor
Shpk	sedoheptulokinase

RNA-seq

Class	Control_1	Control_2	TN_1	TN_2	CBZ_1	CBZ_2	Control_1	Control_2
protein_coding	0.12	0.13	33.36	19.04	72.76	75.76	0.00	0.38
protein_coding	15.56	27.52	10.47	5.86	1.27	4.59	2.64	0.37
protein_coding	0.51	0.14	7.37	20.04	42.51	41.01	0.02	3.02
protein_coding	0.52	0.15	5.71	9.84	16.16	23.18	0.04	3.51
protein_coding	0.15	0.53	6.12	6.49	9.02	28.06	0.05	7.39
protein_coding	0.21	0.37	7.49	3.64	15.45	25.02	0.05	3.69
protein_coding	29.22	71.34	0.37	2.04	1.25	3.85	0.02	11.65
protein_coding	8.81	5.55	3.57	3.24	1.11	1.42	2.11	2.41
protein_coding	0.24	0.38	3.41	3.26	4.12	11.65	0.09	1.52
protein_coding	0.39	0.78	3.82	2.82	11.28	13.98	0.18	2.32
protein_coding	0.35	0.12	2.12	3.21	10.81	6.37	0.09	0.08
protein_coding	1.29	1.88	3.84	3.32	8.55	11.46	0.44	0.39
protein_coding	0.78	0.37	2.23	2.93	9.43	5.09	0.22	1.27
protein_coding	0.13	0.38	0.67	4.03	14.16	4.93	0.11	1.36
protein_coding	2.65	2.93	6.79	5.78	14.39	17.83	0.44	8.65
protein_coding	45.35	43.84	5.86	9.39	22.30	20.72	2.07	1.63
protein_coding	0.80	0.42	9.88	8.85	28.66	25.21	0.07	0.89
protein_coding	0.37	0.43	2.26	1.85	7.63	6.60	0.20	0.55
protein_coding	0.37	0.55	3.08	1.94	4.29	12.30	0.18	2.46
protein_coding	0.75	0.14	8.70	5.11	25.75	21.62	0.06	1.51
protein_coding	0.19	0.87	1.78	1.39	3.72	4.12	0.33	7.58
protein_coding	3.13	3.83	1.11	1.65	0.37	0.31	2.52	9.62
protein_coding	1.12	0.78	17.52	1.22	37.39	40.12	0.10	7.76
protein_coding	3.15	4.70	42.48	0.53	2.20	3.98	0.10	10.13
protein_coding	2.79	2.43	5.22	1.74	7.22	3.92	0.07	3.12
protein_coding	1.26	0.12	2.46	1.91	5.91	7.83	0.32	11.17
protein_coding	1.13	0.87	2.88	15.10	38.14	31.49	0.11	12.76
protein_coding	53.37	49.93	16.61	18.76	5.65	5.13	2.92	1.01
protein_coding	0.82	0.53	2.12	0.75	1.80	7.94	0.47	13.81
protein_coding	0.73	0.64	1.33	1.52	1.41	4.91	0.48	4.70
protein_coding	0.37	0.51	0.33	2.32	7.46	5.94	0.33	2.43
protein_coding	7.05	4.23	42.93	26.72	92.95	103.47	0.16	1.97
protein_coding	0.21	1.21	1.98	0.67	3.34	5.19	0.54	13.92
protein_coding	0.14	4.12	4.54	5.68	13.14	14.31	0.42	1.41
protein_coding	0.35	0.21	1.63	1.01	5.19	4.95	0.21	2.95
protein_coding	6.06	3.87	11.92	13.81	22.78	34.64	0.39	1.37
protein_coding	5.07	5.82	0.73	0.36	2.30	0.78	0.35	0.34
protein_coding	0.83	0.38	1.85	0.54	4.05	10.22	0.51	3.80
protein_coding	1.87	2.24	23.77	20.28	46.99	55.09	0.09	1.31
protein_coding	3.64	4.59	36.95	51.31	2.05	9.09	0.09	17.56
protein_coding	0.67	0.87	0.91	0.61	1.69	2.49	1.02	0.65
protein_coding	6.33	0.93	9.84	57.62	333.16	361.44	0.11	45.33
protein_coding	70.23	70.25	33.09	33.39	15.32	16.13	2.11	5.97
protein_coding	1.37	2.35	1.40	0.61	2.18	4.81	1.85	3.98
protein_coding	0.34	0.37	1.56	0.27	3.84	10.30	0.39	1.64
protein_coding	3.80	2.77	20.16	17.56	40.71	42.24	0.17	2.18
protein_coding	0.82	0.73	1.15	0.65	1.51	3.96	0.86	3.84
protein_coding	7.26	0.84	31.70	25.93	72.37	82.59	0.14	2.31
protein_coding	14.94	21.13	6.85	8.40	0.32	3.86	2.37	0.67
protein_coding	1.49	1.06	10.36	8.69	23.34	23.33	0.13	5.68

protein_coding	0.32	0.37	1.15	0.34	3.29	2.06	0.47	3.20
protein_coding	366.13	341.39	93.04	126.58	5.68	6.89	3.22	3.80
protein_coding	0.10	0.44	1.06	0.38	2.66	1.99	0.37	0.82
protein_coding	7.14	5.18	29.19	54.49	126.86	129.07	0.15	7.26
protein_coding	1.87	1.63	19.81	3.50	45.38	33.87	0.15	2.77
protein_coding	1.11	0.37	5.22	2.79	13.72	9.38	0.18	1.36
protein_coding	18.15	23.26	8.78	10.35	3.86	3.73	2.16	5.85
protein_coding	6.19	5.07	22.96	51.02	150.48	123.77	0.15	9.17
protein_coding	18.64	19.69	141.48	109.07	352.90	378.07	0.15	0.53
protein_coding	3.99	3.98	8.49	10.17	18.51	23.65	0.43	39.23
protein_coding	5.25	5.85	45.31	26.65	320.17	333.82	0.15	2.76
protein_coding	10.99	8.43	4.28	4.81	1.21	1.63	2.14	6.28
protein_coding	2.66	0.35	6.28	11.26	30.24	38.27	0.17	4.44
protein_coding	19.84	16.07	8.60	2.93	1.11	1.70	3.11	5.37
protein_coding	0.24	0.37	0.71	0.53	2.25	1.14	0.49	2.86
protein_coding	3.30	9.30	38.51	39.23	90.71	100.74	0.16	2.66
protein_coding	0.37	0.71	0.73	0.44	3.05	2.49	0.92	19.84
protein_coding	0.37	1.07	1.27	5.13	11.14	13.99	0.22	0.24
protein_coding	4.43	1.82	14.33	18.37	56.01	56.97	0.19	13.72
protein_coding	0.54	0.95	0.75	0.84	2.64	12.02	0.94	6.69
protein_coding	2.96	3.64	7.59	7.50	14.62	17.83	0.44	2.53
protein_coding	0.60	0.83	2.08	1.22	8.30	1.26	0.43	2.49
protein_coding	22.21	64.65	19.27	19.41	7.83	6.83	2.25	5.55
protein_coding	3.85	0.65	6.20	10.70	23.88	23.43	0.27	0.89
protein_coding	0.45	0.46	1.18	0.54	4.34	1.63	0.53	5.38
protein_coding	14.64	14.97	52.90	66.56	132.27	143.59	0.25	4.50
protein_coding	2.23	2.25	8.46	9.28	22.42	22.38	0.25	4.14
protein_coding	2.83	1.70	9.35	6.78	38.49	42.61	0.28	7.51
protein_coding	83.18	71.83	32.75	32.92	12.88	17.47	2.36	2.28
protein_coding	8.60	2.85	26.85	15.56	47.34	65.55	0.27	0.47
protein_coding	42.88	39.73	19.94	19.17	9.46	8.29	2.11	3.69
protein_coding	1.20	1.33	4.02	5.17	11.76	11.63	0.28	1.84
protein_coding	25.04	30.66	10.49	7.98	2.81	4.64	3.01	4.65
protein_coding	3.35	2.37	12.44	8.05	35.79	37.72	0.28	0.54
protein_coding	2.99	2.73	9.91	9.35	20.41	24.07	0.30	2.62
protein_coding	0.35	0.54	0.29	0.55	0.71	1.71	1.06	1.47
protein_coding	1.34	1.88	3.60	7.47	13.73	17.64	0.29	1.86
protein_coding	0.54	4.11	6.15	8.09	17.95	21.23	0.33	1.63
protein_coding	15.22	16.55	7.87	7.94	2.82	4.74	2.01	6.88
protein_coding	1.12	2.85	4.97	8.45	25.14	23.22	0.30	7.52
protein_coding	0.54	0.91	1.82	1.47	4.46	10.97	0.44	8.60
protein_coding	24.31	10.68	3.83	5.39	0.76	2.31	3.80	3.54
protein_coding	0.54	4.48	7.89	6.97	18.65	17.68	0.34	2.75
protein_coding	2.98	1.24	6.05	7.13	10.39	19.76	0.32	2.68
protein_coding	2.82	2.73	9.28	7.96	19.73	23.94	0.32	30.92
protein_coding	9.85	3.02	13.76	15.94	45.44	31.11	0.43	2.85
protein_coding	7.52	8.42	30.83	17.90	69.92	70.63	0.33	4.50
protein_coding	3.79	2.69	9.81	9.48	8.71	33.11	0.34	1.33
protein_coding	3.09	3.82	11.44	19.08	39.45	43.68	0.23	1.17
protein_coding	1.70	1.99	5.81	5.11	12.53	14.84	0.34	5.81
protein_coding	1.32	0.65	1.57	2.63	7.16	8.00	0.47	2.04
protein_coding	7.64	3.03	14.17	12.42	38.34	30.75	0.40	0.98

protein_coding	1.91	4.83	7.36	7.99	46.04	42.48	0.44	4.02
protein_coding	2.74	4.41	10.44	9.97	21.15	20.60	0.35	11.27
protein_coding	0.87	1.23	1.37	2.28	9.09	3.43	0.58	4.22
protein_coding	12.36	13.99	6.07	3.85	0.39	0.88	2.66	5.29
protein_coding	1.46	7.60	13.72	11.15	29.97	30.27	0.36	4.58
protein_coding	6.85	5.34	14.66	18.71	39.75	53.07	0.37	17.47
protein_coding	0.74	6.07	7.17	9.49	21.59	22.78	0.41	10.23
protein_coding	1.82	2.92	5.90	6.73	15.69	15.84	0.38	0.10
protein_coding	5.05	5.03	14.92	17.51	42.06	32.14	0.31	4.65
protein_coding	50.34	45.12	117.99	135.32	314.03	237.66	0.38	1.69
protein_coding	1.27	1.19	3.56	2.90	8.23	6.19	0.38	23.79
protein_coding	17.87	13.28	7.01	3.44	0.88	2.18	2.98	1.44
protein_coding	0.45	0.55	2.39	1.01	5.10	7.95	0.29	1.48
protein_coding	3.29	1.82	8.17	5.11	18.51	17.29	0.38	6.56
protein_coding	4.80	5.31	10.51	15.71	59.58	59.17	0.39	0.10
protein_coding	6.61	6.98	2.68	1.59	0.54	0.97	3.17	8.43
protein_coding	3.86	3.01	10.88	6.87	33.56	35.35	0.39	6.21
protein_coding	11.89	5.92	22.30	22.49	41.22	49.14	0.40	3.90
protein_coding	4.44	3.53	10.69	9.32	19.04	29.48	0.40	9.22
protein_coding	8.05	5.67	15.76	18.59	38.80	38.29	0.40	5.73
protein_coding	0.37	2.06	5.43	2.91	16.88	10.07	0.29	6.98
protein_coding	0.87	0.75	0.22	0.28	0.51	0.74	3.25	1.75
protein_coding	14.37	21.38	43.38	44.99	103.04	79.32	0.40	1.34
protein_coding	7.16	4.77	17.44	28.48	62.70	68.20	0.26	2.63
protein_coding	18.10	14.07	40.66	38.06	74.48	88.14	0.41	10.28
protein_coding	0.77	0.79	0.29	0.71	0.87	0.60	1.55	10.44
protein_coding	6.03	2.29	9.73	10.20	24.38	19.13	0.42	3.37
protein_coding	522.46	487.72	24.55	168.79	13.91	10.65	5.22	5.52
protein_coding	0.42	0.73	0.77	2.18	5.95	2.79	0.39	3.42
protein_coding	1.97	7.74	12.55	10.56	29.46	21.24	0.42	2.23
protein_coding	13.62	11.40	29.91	29.17	59.63	69.46	0.42	4.13
protein_coding	35.58	35.39	77.23	88.72	246.14	242.34	0.43	7.01
protein_coding	22.74	27.32	12.17	12.45	8.07	3.46	2.03	17.90
protein_coding	1.29	1.37	0.32	0.21	0.74	0.73	4.99	5.20
protein_coding	7.32	5.33	1.29	1.05	0.32	0.77	5.42	7.48
protein_coding	26.07	17.83	7.90	10.85	5.44	2.32	2.34	135.90
protein_coding	2.82	3.29	6.15	7.78	16.40	14.88	0.44	24.42
protein_coding	1.56	0.55	2.17	2.12	4.96	5.00	0.49	22.53
protein_coding	0.76	0.67	1.94	0.61	4.59	5.18	0.56	11.09
protein_coding	3.67	3.31	7.34	8.32	22.77	22.61	0.45	55.75
protein_coding	0.35	0.75	2.12	0.81	6.07	11.82	0.38	75.61
protein_coding	6.44	0.78	6.93	7.42	18.18	16.64	0.50	195.66
protein_coding	4.99	7.33	37.28	25.23	103.16	114.85	0.20	56.13
protein_coding	3.00	8.33	11.63	13.21	31.03	26.18	0.46	30.82
protein_coding	1.71	1.58	10.99	14.38	35.86	37.67	0.13	14.51
protein_coding	0.97	2.23	2.93	3.46	9.18	7.53	0.50	36.99
protein_coding	1.32	0.87	2.23	2.43	4.06	7.22	0.47	190.37
protein_coding	27.27	36.41	15.86	15.87	5.42	10.23	2.01	45.25
protein_coding	8.23	10.74	3.17	2.33	0.68	0.53	3.45	19.33
protein_coding	0.86	1.07	0.26	0.23	0.61	0.83	3.97	18.64
protein_coding	45.25	29.17	18.43	18.25	9.07	7.81	2.03	30.63
protein_coding	0.43	0.43	4.20	7.88	11.78	20.37	0.07	57.69

protein_coding	27.43	21.87	12.02	12.14	5.61	5.45	2.04	25.45
protein_coding	16.79	17.02	8.23	8.32	4.73	3.42	2.04	4.11
protein_coding	10.67	8.82	4.68	4.86	1.33	2.87	2.04	11.50
protein_coding	11.30	10.93	3.12	1.64	0.78	1.20	4.66	57.30
protein_coding	39.83	32.44	17.40	17.74	8.75	6.07	2.06	33.88
protein_coding	133.82	110.03	58.43	58.27	26.74	26.46	2.09	20.78
protein_coding	163.61	183.20	46.41	76.63	14.81	10.20	2.82	4.23
protein_coding	5.01	3.40	1.86	2.00	0.76	1.08	2.18	21.12
protein_coding	398.48	354.06	81.56	112.01	0.61	1.53	3.89	2.47
protein_coding	48.46	54.98	24.66	23.97	8.35	13.29	2.13	187.37
protein_coding	29.10	28.59	13.03	13.78	5.07	6.51	2.15	75.56
protein_coding	36.99	42.18	17.99	18.71	6.58	11.00	2.16	72.34
protein_coding	56.19	59.80	6.48	6.95	3.27	2.75	8.63	366.00
protein_coding	79.17	85.14	36.90	38.03	19.00	16.55	2.19	18.82
protein_coding	7.03	6.40	40.82	43.70	105.87	89.11	0.16	19.58
protein_coding	19.29	12.48	7.62	6.99	6.73	0.87	2.17	30.71
protein_coding	0.45	0.74	0.52	1.89	2.94	8.24	0.50	37.46
protein_coding	41.84	41.18	18.63	19.53	10.46	7.14	2.18	9.89
protein_coding	0.37	0.53	0.89	2.27	12.80	10.51	0.29	43.45
protein_coding	28.96	33.95	14.07	14.60	6.07	7.81	2.19	12.57
protein_coding	2058.01	2271.99	1038.72	929.27	0.77	9.81	2.20	55.88
protein_coding	0.71	0.63	0.91	0.32	4.00	1.56	1.09	23.70
protein_coding	32.85	27.00	13.06	14.01	5.73	2.59	2.21	20.42
protein_coding	74.95	74.01	32.14	35.24	13.65	12.73	2.21	33.02
protein_coding	19.42	13.92	4.59	6.38	1.70	2.65	3.04	44.11
protein_coding	53.37	49.93	21.83	24.29	5.65	5.13	2.24	17.12
protein_coding	2.90	3.45	16.59	28.54	81.93	110.73	0.14	22.02
protein_coding	25.59	16.48	10.06	8.49	4.12	3.91	2.27	2.27
protein_coding	5.44	13.59	4.31	4.03	0.73	1.91	2.28	30.46
protein_coding	4.38	3.79	9.19	13.50	41.62	40.80	0.36	134.28
protein_coding	79.17	51.55	27.87	29.36	13.07	11.49	2.28	13.13
protein_coding	92.77	104.28	39.81	45.53	21.87	15.20	2.31	165.89
protein_coding	1.62	1.89	0.68	0.83	0.21	0.43	2.31	10.78
protein_coding	18.28	14.68	7.16	7.11	3.62	3.23	2.31	30.44
protein_coding	8.17	9.42	3.28	4.31	20.72	10.51	2.32	12.95
protein_coding	19.94	15.18	6.96	8.14	2.11	4.26	2.33	1003.78
protein_coding	64.40	61.78	27.92	25.86	11.38	14.24	2.35	34.76
protein_coding	3.21	1.31	1.63	1.79	6.15	10.13	1.32	24.56
protein_coding	9.85	13.27	5.09	4.74	2.13	1.23	2.35	27.27
protein_coding	2.50	3.80	1.32	1.35	4.23	2.13	2.36	57.04
protein_coding	106.42	114.10	22.53	55.13	1.49	5.24	2.84	79.13
protein_coding	41.89	42.45	15.03	20.30	7.69	6.94	2.39	6.00
protein_coding	22.85	35.23	11.73	12.52	3.41	8.14	2.39	6.67
protein_coding	3.80	79.88	16.58	18.35	4.09	7.16	2.40	21.36
protein_coding	0.46	0.43	6.10	12.00	33.52	17.57	0.05	6.91
protein_coding	3.93	2.35	1.04	1.57	1.25	1.37	2.41	48.43
protein_coding	41.04	42.32	16.71	17.83	1.51	0.92	2.41	29.45
protein_coding	8.78	9.02	4.03	3.33	2.79	1.58	2.42	811.44
protein_coding	34.36	35.42	13.95	14.71	6.23	6.59	2.43	31.08
protein_coding	1.89	2.40	3.72	0.95	8.23	13.49	0.92	104.47
protein_coding	12.35	27.36	7.26	9.01	2.27	4.22	2.44	42.31
protein_coding	0.98	3.97	1.12	0.91	0.30	0.10	2.44	93.55

protein_coding	31.90	40.29	12.83	16.74	4.41	2.98	2.44	27.96
protein_coding	38.87	43.90	16.60	17.08	5.35	7.64	2.46	6.43
protein_coding	34.05	42.87	3.65	4.95	2.39	0.94	8.95	22.69
protein_coding	44.81	53.30	18.46	21.41	7.83	9.07	2.46	29.41
protein_coding	16.79	19.38	3.81	7.60	1.53	3.29	3.17	1.84
protein_coding	24.76	32.05	12.07	10.52	2.16	2.54	2.52	37.09
protein_coding	34.36	35.42	13.29	14.24	6.23	6.59	2.53	15.97
protein_coding	5.60	2.56	1.25	1.96	0.70	0.42	2.54	15.38
protein_coding	7.25	6.72	1.01	1.34	0.01	1.40	5.95	18.92
protein_coding	40.94	30.12	10.40	17.60	5.91	3.66	2.54	36.32
protein_coding	62.06	50.20	27.76	16.33	8.83	4.49	2.55	610.73
protein_coding	123.57	124.84	41.75	54.98	18.65	18.52	2.57	11.02
protein_coding	2.71	0.68	4.72	3.77	7.80	12.82	0.40	43.76
protein_coding	8.05	8.83	4.10	2.48	0.05	1.07	2.57	14.82
protein_coding	107.79	116.62	44.73	41.63	1.39	5.36	2.60	44.81
protein_coding	0.87	1.07	0.47	0.27	2.64	2.67	2.63	31.67
protein_coding	177.04	213.91	32.80	42.90	12.28	14.75	5.16	67.93
protein_coding	64.93	64.36	30.61	25.75	9.46	8.86	2.29	21.44
protein_coding	1.37	2.51	0.56	0.89	1.70	2.40	2.67	661.19
protein_coding	99.83	85.67	37.04	31.10	2.80	1.99	2.72	2.30
protein_coding	14.09	12.74	5.84	4.37	2.62	0.82	2.63	28.77
protein_coding	2.18	2.22	0.99	0.62	3.55	1.33	2.73	11.45
protein_coding	82.65	84.98	34.90	26.14	12.09	8.36	2.75	24.27
protein_coding	7.46	6.05	3.06	1.79	0.31	0.85	2.78	154.81
protein_coding	13.76	12.61	6.00	5.28	2.08	1.31	2.34	34.54
protein_coding	13.18	27.90	9.63	5.00	3.05	0.91	2.81	28.93
protein_coding	62.72	52.54	19.51	21.44	7.23	5.87	2.81	380.42
protein_coding	13.62	11.68	5.07	3.90	1.78	0.71	2.82	33.51
protein_coding	0.01	0.54	0.53	0.68	2.41	1.09	0.45	64.83
protein_coding	23.40	22.04	8.63	7.26	3.19	2.59	2.86	45.76
protein_coding	4.64	4.55	13.16	13.56	27.68	28.53	0.34	69.78
protein_coding	1.98	4.16	1.13	1.02	0.85	1.33	2.86	40.94
protein_coding	1.10	2.66	7.75	4.47	13.13	20.85	0.31	0.70
protein_coding	88.85	102.03	38.17	27.68	10.85	13.49	2.90	31.08
protein_coding	5.80	0.78	0.67	0.32	3.25	1.92	6.65	2.88
protein_coding	9.46	10.21	3.72	2.98	1.70	0.95	2.94	24.83
protein_coding	61.66	42.59	14.68	20.37	7.00	3.58	2.97	0.96
protein_coding	18.10	14.07	38.06	32.17	74.48	88.14	0.46	41.78
protein_coding	91.68	130.75	28.79	44.29	10.95	15.46	3.04	17.28
protein_coding	24.62	20.81	9.25	7.05	3.59	3.08	2.79	51.73
protein_coding	176.88	189.71	53.03	62.30	7.59	8.37	3.18	5.06
protein_coding	29.09	20.12	0.60	10.60	3.25	0.26	4.39	67.45
protein_coding	129.85	141.48	44.71	38.29	2.43	2.47	3.27	63.79
protein_coding	3.30	5.46	15.17	17.12	44.98	44.67	0.27	33.42
protein_coding	2.26	1.85	0.91	0.33	1.86	1.36	3.30	18.98
protein_coding	4.83	11.75	2.13	2.77	0.83	2.38	3.38	4.19
protein_coding	6.86	4.67	1.90	1.51	1.37	0.08	3.38	100.89
protein_coding	3.70	2.70	1.11	0.74	2.63	0.10	3.45	1.67
protein_coding	49.80	37.55	14.23	8.44	1.34	2.75	3.85	161.72
protein_coding	6.47	0.00	8.20	12.21	29.02	21.84	0.32	8.46
protein_coding	32.90	24.69	7.06	9.30	0.82	3.60	3.52	26.36
protein_coding	8.84	13.84	2.66	3.71	0.08	2.14	3.56	3.08

protein_coding	3.14	0.01	8.00	3.52	17.26	22.33	0.27	1.63
protein_coding	16.82	15.21	4.67	4.32	0.71	1.65	3.56	214.95
protein_coding	4.28	6.84	0.41	2.67	0.53	0.33	3.60	355.12
protein_coding	1222.21	1274.04	582.30	587.74	280.84	298.73	2.13	36.48
protein_coding	55.49	37.21	6.72	18.03	2.10	4.27	3.74	12.07
protein_coding	78.30	77.79	21.09	20.26	9.74	8.85	3.78	6.52
protein_coding	1.92	2.37	0.64	0.27	2.32	3.25	4.68	4.03
protein_coding	5.94	5.11	1.72	1.19	1.51	1.68	3.80	13.02
protein_coding	8.74	12.15	3.69	2.05	4.35	3.58	3.65	17.11
protein_coding	166.55	162.34	25.49	59.01	9.41	9.63	3.89	20.16
protein_coding	61.12	71.75	9.45	23.36	2.68	2.13	4.05	12.61
protein_coding	194.90	176.19	30.69	58.47	7.68	5.04	4.16	3.80
protein_coding	0.05	0.65	0.81	1.26	3.61	1.80	0.34	25.59
protein_coding	78.89	71.93	18.96	15.21	5.55	5.91	4.41	2.89
protein_coding	78.89	72.51	11.46	19.54	5.55	5.91	4.88	26.12
protein_coding	1.56	6.16	1.19	0.38	0.09	0.63	4.93	172.61
protein_coding	8.10	12.15	2.98	0.99	2.55	3.26	5.10	2.08
protein_coding	8.76	5.33	3.12	3.24	1.40	1.72	2.22	2.89
protein_coding	54.33	55.52	1.98	12.38	1.37	0.61	7.65	45.23
protein_coding	3.20	6.26	1.48	0.24	12.65	1.35	5.48	2.08
protein_coding	4.54	17.04	1.10	2.65	0.05	0.81	5.76	3.45
protein_coding	9.91	13.51	5.35	5.48	2.82	2.00	2.16	291.57
protein_coding	5.15	2.11	0.79	0.40	3.59	19.83	6.10	67.71
protein_coding	60.62	68.78	32.35	26.31	11.25	2.50	2.21	2.68
protein_coding	9.02	4.44	1.56	0.59	0.83	4.43	6.28	3.21
protein_coding	18.46	20.94	9.06	9.52	3.71	4.53	2.12	34.99
protein_coding	11.69	7.10	1.30	0.93	11.35	2.78	8.40	7.44
protein_coding	0.09	2.32	1.94	0.90	6.29	2.10	0.85	136.57
protein_coding	3.56	5.46	0.87	0.06	0.05	0.27	9.70	8.60
protein_coding	69.62	63.59	30.62	29.02	4.18	2.88	2.23	4.78

H3K9ac ChIP-seq						HDAC3 ChIP-seq		
TN_1	TN_2	CBZ_1	CBZ_2	Control_1	Control_2	TN_1	TN_2	CBZ_1
0.15	3.74	19.65	47.91	68.44	0.02	7.25	14.20	1.04
10.81	7.40	7.25	18.22	11.99	0.76	91.25	74.25	4.36
2.71	10.59	0.36	8.04	12.48	0.03	5.39	5.19	0.25
8.34	6.51	5.61	15.77	14.49	0.98	3.56	2.59	0.34
3.03	2.54	3.61	3.56	16.36	1.69	0.34	0.62	1.95
37.39	4.08	1.43	10.50	8.85	7.45	2.20	6.11	0.51
0.14	1.05	5.24	11.88	9.06	1.87	7.22	7.34	0.62
2.54	2.18	3.10	8.57	6.11	0.94	11.51	9.43	2.36
1.41	4.35	0.75	13.92	8.27	0.58	14.16	16.37	2.17
7.46	2.40	2.33	6.54	7.73	2.07	8.40	2.57	4.92
0.15	2.98	1.49	6.92	7.88	0.05	4.17	2.68	4.74
1.99	1.05	2.89	19.15	9.39	0.60	0.15	3.36	5.21
2.67	2.58	1.02	7.17	8.18	1.10	3.51	5.04	1.31
1.40	1.64	1.90	5.46	8.14	0.78	7.39	21.62	3.65
2.19	0.78	2.73	14.30	9.08	3.09	3.69	6.08	0.71
3.82	0.54	2.96	6.99	4.84	1.56	1.27	1.66	0.28
0.30	2.46	0.98	2.13	13.92	0.35	2.41	3.76	0.94
2.66	0.72	2.71	10.81	10.50	0.93	6.55	8.09	1.65
3.66	2.03	1.25	4.78	6.88	1.86	1.52	3.23	0.88
1.98	18.84	2.90	0.88	4.36	0.06	4.84	3.53	0.39
2.35	1.57	1.51	4.12	2.51	3.23	1.48	1.66	4.59
6.98	3.76	4.62	3.96	6.54	0.07	9.80	7.98	2.28
4.49	1.03	1.92	6.79	3.64	4.15	1.37	1.18	0.22
8.49	1.30	1.47	6.15	5.95	6.74	0.37	1.65	4.44
8.32	2.14	0.60	6.35	3.10	43.47	1.58	3.03	0.39
11.06	1.11	1.41	2.58	4.17	8.81	3.23	2.81	2.21
11.40	1.01	1.51	8.34	4.66	9.60	14.77	10.37	4.79
2.18	1.35	1.15	5.39	5.23	1.27	2.83	3.07	0.67
3.84	1.89	0.53	7.76	5.79	7.31	1.10	1.31	4.15
0.80	3.80	5.82	11.17	14.33	0.57	3.21	3.65	4.14
1.98	0.54	1.56	3.82	2.51	2.10	0.91	1.46	4.33
4.54	15.81	5.66	57.03	68.30	0.30	58.14	62.31	5.67
20.94	0.48	0.27	4.48	4.50	0.10	2.98	14.17	3.98
33.30	1.44	0.20	2.83	6.37	21.11	8.94	8.45	3.16
19.57	12.21	11.65	22.81	26.04	0.94	11.07	22.13	3.34
2.13	2.49	14.84	32.15	28.25	0.20	3.36	2.39	1.31
0.35	0.72	0.82	1.91	2.00	0.45	68.97	50.00	7.29
6.06	1.31	0.20	1.69	6.09	6.55	1.39	4.42	0.63
0.73	4.18	6.22	26.86	15.00	0.20	1.01	1.47	3.92
2.35	0.64	0.81	3.50	1.88	13.72	0.42	0.60	1.70
0.37	0.91	0.53	2.04	3.39	0.71	9.31	10.62	4.49
71.90	2.63	2.00	3.52	1.51	0.15	0.37	1.41	3.88
0.10	7.18	33.30	62.18	71.02	0.15	4.21	9.80	2.32
1.11	14.25	19.57	37.43	40.85	0.15	6.26	11.14	1.76
1.06	3.79	7.66	15.73	14.93	0.24	21.99	26.44	3.47
0.37	0.89	0.42	1.06	3.01	1.96	1.47	1.30	3.44
2.24	10.10	5.11	18.90	26.67	0.40	0.60	2.14	3.44
0.10	10.29	4.81	34.25	42.55	0.16	12.34	21.95	7.89
5.22	0.65	0.60	3.79	6.76	4.73	10.25	8.25	1.88
2.46	0.60	0.65	1.52	1.65	6.53	43.49	46.12	18.77

8.04	13.51	55.58	129.96	137.83	0.16	13.41	13.01	3.77
1.02	0.59	0.62	0.85	1.78	3.98	1.41	1.11	3.36
0.11	0.75	0.46	0.93	3.72	0.77	4.68	2.60	3.34
2.11	0.25	0.91	1.34	2.41	8.15	4.50	4.05	1.24
2.84	20.20	11.58	39.32	48.13	0.18	3.65	1.57	3.31
4.16	13.47	17.77	39.08	47.92	0.18	0.93	0.71	0.33
11.63	52.10	46.83	115.90	101.52	0.18	18.94	18.05	3.30
23.26	41.24	10.05	84.73	105.19	0.63	2.32	5.30	1.04
5.07	0.48	0.58	2.48	2.26	5.27	9.19	50.97	18.48
0.58	0.49	0.54	3.01	1.10	38.81	12.53	8.28	4.13
0.54	10.64	3.83	37.90	37.09	0.23	43.55	35.10	13.38
3.83	0.20	0.78	1.53	2.41	10.35	27.25	23.76	10.35
0.37	20.22	27.87	57.15	66.25	0.10	24.83	12.99	3.24
5.00	26.93	22.38	59.60	63.02	0.21	21.95	35.31	11.50
3.30	16.01	12.03	30.44	35.29	0.22	5.34	49.64	17.07
1.12	2.50	3.03	9.81	9.84	0.68	41.17	36.32	15.90
0.54	0.21	0.69	1.88	1.07	22.58	38.48	44.10	13.30
1.88	0.47	0.40	1.06	0.95	2.42	6.17	5.20	2.23
5.22	12.15	10.89	26.67	29.81	0.82	24.69	18.74	3.23
8.81	25.94	37.95	85.23	79.11	0.24	6.03	31.26	15.63
3.06	12.44	9.74	32.90	30.13	0.25	19.66	72.40	34.04
2.12	8.21	10.01	18.86	26.73	0.25	25.30	22.27	8.90
4.08	20.49	17.15	102.66	99.70	0.26	9.91	7.47	3.16
3.05	1.64	2.21	6.71	4.30	1.02	41.89	42.45	14.64
5.48	12.11	29.67	74.71	91.64	0.26	1.45	1.15	0.58
7.02	23.73	20.24	47.96	46.96	0.26	46.06	58.48	18.97
5.58	16.08	20.31	32.24	52.78	0.27	5.64	102.03	39.61
0.10	14.04	13.50	29.30	36.13	0.28	15.60	15.77	2.51
0.69	1.27	1.56	3.62	3.63	1.05	7.36	5.23	2.25
11.82	24.11	17.43	60.68	43.87	0.30	4.71	6.76	2.07
3.50	11.27	13.58	31.89	31.63	0.29	21.44	24.20	9.24
0.74	2.58	6.30	16.14	9.38	0.29	4.03	2.42	2.49
2.36	12.36	10.95	43.50	39.76	0.30	7.58	4.52	2.49
4.06	4.28	9.22	20.45	20.40	0.34	10.85	13.49	2.48
1.70	8.13	5.88	17.44	18.97	0.31	47.29	43.06	2.48
4.97	3.39	1.67	7.92	8.55	1.27	1.82	2.54	0.73
1.82	3.26	2.92	7.04	9.32	0.60	3.15	0.32	2.47
3.83	0.29	0.34	1.38	8.39	8.69	13.18	12.97	6.50
1.46	12.42	13.53	30.52	27.06	0.32	9.86	28.71	8.05
8.42	19.47	28.87	69.92	70.63	0.33	13.18	11.91	2.40
2.85	0.39	0.21	0.59	2.11	18.97	7.10	13.64	2.44
2.85	11.18	7.85	23.19	30.84	0.34	7.89	5.88	2.77
1.45	7.76	4.49	17.71	17.96	0.34	6.56	9.53	2.35
3.76	10.13	8.49	21.03	20.78	0.35	13.72	16.18	6.27
10.89	64.71	54.31	42.17	261.08	0.35	7.96	6.95	3.73
5.10	11.17	11.06	24.96	27.90	0.36	17.02	11.97	5.61
4.17	12.76	11.40	39.22	33.60	0.36	2.22	4.64	1.38
4.02	0.36	0.20	4.65	6.31	9.64	2.93	2.15	2.31
1.21	2.81	3.80	8.69	9.24	0.36	0.40	0.89	2.31
8.99	16.86	23.81	47.84	48.15	0.36	8.13	2.45	2.30
1.61	4.95	5.04	7.98	15.14	0.36	3.74	6.41	2.30
0.91	2.47	2.65	5.92	4.88	0.37	8.54	7.64	2.29

0.10	6.08	4.89	14.04	10.81	0.38	12.28	20.74	2.28
7.57	31.10	18.85	72.01	92.34	0.38	18.03	14.61	7.25
5.10	11.32	13.01	38.76	28.68	0.38	11.03	11.71	5.55
4.29	12.03	12.79	26.19	27.12	0.39	6.96	6.36	2.95
6.67	12.45	16.40	42.21	36.43	0.39	21.51	8.63	6.47
14.90	40.77	42.11	91.92	103.37	0.39	19.11	7.73	12.82
8.38	26.98	20.39	67.99	56.18	0.39	8.99	10.83	31.03
1.71	2.20	2.39	5.13	6.66	0.39	3.09	0.77	29.30
3.05	10.12	9.33	18.45	24.22	0.40	2.05	0.39	7.83
5.00	8.14	8.70	23.00	25.73	0.40	14.83	18.44	8.32
17.87	49.27	54.98	120.86	139.01	0.40	12.88	17.47	2.23
3.24	5.74	5.85	12.88	11.20	0.40	9.11	7.98	4.08
0.10	1.95	1.96	4.61	5.28	0.40	13.40	2.77	2.51
5.19	15.36	13.51	28.16	36.09	0.41	10.37	9.45	4.62
2.05	3.00	2.27	10.37	10.16	0.41	79.17	85.14	38.93
0.10	9.95	10.77	31.89	12.19	0.41	23.70	23.42	10.15
3.48	10.82	12.53	28.83	23.83	0.41	36.58	40.18	16.00
2.09	7.52	6.88	25.72	23.32	0.42	30.46	30.19	13.39
10.07	24.32	21.94	53.68	50.88	0.42	89.70	35.90	28.51
7.79	15.99	16.22	36.32	32.89	0.42	9.68	9.62	4.35
11.20	19.06	24.17	52.46	48.91	0.42	19.64	21.92	10.32
1.37	4.19	3.20	12.85	8.06	0.42	5.33	2.75	1.95
1.79	4.10	3.17	4.73	11.94	0.43	41.17	42.04	18.59
2.90	6.90	5.93	14.90	13.20	0.43	5.83	3.00	2.19
5.51	15.78	20.52	136.01	159.22	0.43	6.91	6.49	2.19
5.51	18.92	17.57	61.13	55.64	0.44	6.52	6.09	2.18
5.27	10.65	9.06	21.07	22.64	0.44	12.28	20.74	2.17
4.42	10.31	12.28	22.02	29.79	0.44	5.20	6.51	2.81
3.54	7.22	8.44	21.34	16.90	0.44	20.83	26.11	10.98
3.65	7.30	5.95	7.08	22.47	0.44	30.29	37.45	16.26
0.10	4.34	5.13	10.35	10.86	0.45	36.98	41.96	19.30
5.14	13.41	12.62	38.55	40.89	0.47	30.25	33.46	15.82
18.48	37.68	39.84	93.15	94.75	0.47	16.69	14.80	7.82
3.35	8.55	9.23	76.39	90.61	0.48	10.76	13.13	5.71
7.88	16.03	15.42	31.26	33.80	0.49	13.31	19.23	8.07
173.65	316.27	309.55	797.21	880.11	0.49	69.86	77.38	36.36
22.91	11.83	11.83	3.26	8.57	2.00	23.10	22.97	11.34
17.38	9.97	9.97	6.02	3.95	2.00	34.71	31.69	16.24
8.89	4.97	4.93	1.56	3.29	2.02	42.32	36.10	19.26
60.56	29.08	28.23	4.06	4.39	2.03	42.06	37.59	19.82
56.50	31.92	33.03	18.99	12.40	2.03	10.48	8.64	2.02
198.85	96.74	96.66	49.62	45.15	2.04	4.82	11.65	4.08
66.81	30.74	28.66	17.80	10.39	2.07	24.94	19.15	11.01
35.58	16.14	15.91	7.46	3.48	2.07	41.28	37.30	19.64
15.63	7.00	7.54	2.89	3.18	2.07	8.87	0.10	9.25
42.18	18.80	19.30	6.58	11.00	2.08	1.10	1.55	2.76
126.30	77.54	74.29	33.56	29.69	2.09	3.27	0.10	3.57
29.17	18.25	17.41	9.07	7.81	2.09	4.94	3.46	8.65
19.90	9.37	9.40	3.47	5.90	2.09	8.21	8.98	17.65
23.08	9.83	10.11	3.08	5.62	2.09	7.66	4.94	13.58
25.31	13.29	13.26	10.42	2.65	2.11	15.04	17.15	36.20
90.53	33.62	36.67	7.44	10.90	2.11	14.01	19.18	37.89

31.94	14.18	12.99	4.65	8.34	2.11	23.01	20.07	51.21
6.79	2.66	2.50	0.86	1.06	2.11	8.42	8.93	19.41
20.01	7.37	7.51	2.04	3.56	2.12	1.99	2.09	5.03
54.50	25.04	27.50	12.41	10.05	2.13	0.55	1.61	2.53
36.46	17.58	15.37	6.45	7.74	2.13	4.33	1.26	6.14
15.49	7.96	8.91	5.26	2.70	2.15	4.10	4.65	9.51
5.03	2.04	2.24	5.62	1.26	2.16	5.35	2.24	7.69
25.13	10.97	10.38	4.52	2.90	2.17	20.87	27.04	48.89
5.04	1.69	1.75	0.75	0.87	2.18	5.28	10.64	17.78
456.85	670.89	626.00	280.69	314.59	2.19	4.96	5.27	10.22
106.69	41.91	41.18	19.45	17.16	2.19	17.75	24.09	9.79
95.55	41.36	35.17	5.40	6.28	2.19	4.51	1.68	6.70
432.09	178.61	183.96	88.24	87.46	2.20	3.87	6.59	13.32
14.49	7.10	8.00	3.33	3.54	2.21	3.85	2.12	6.09
17.14	8.86	7.75	3.78	3.64	2.21	2.69	0.41	3.14
28.72	13.83	13.04	5.73	6.15	2.21	9.78	9.38	23.51
30.76	14.84	15.97	7.72	6.80	2.21	2.60	2.69	7.31
10.64	4.75	4.49	2.79	1.69	2.22	4.05	2.89	10.11
38.69	19.69	17.15	7.49	9.60	2.23	9.02	3.55	14.25
8.91	4.92	4.70	2.20	2.07	2.23	10.33	0.10	12.84
47.49	22.50	23.75	8.88	6.72	2.23	14.51	10.68	29.71
23.24	9.45	11.51	4.63	3.38	2.24	13.23	8.63	25.15
17.72	8.36	8.63	4.70	2.22	2.24	1.14	1.12	3.23
35.29	16.58	13.77	6.26	7.37	2.25	4.10	3.18	4.07
34.15	19.09	15.59	6.73	5.03	2.26	8.68	4.17	14.77
11.88	6.11	6.62	2.87	3.14	2.28	3.66	3.49	10.30
19.89	8.79	9.53	3.31	4.57	2.29	4.08	3.38	10.70
1.63	0.91	0.78	2.09	1.19	2.30	4.64	4.55	12.96
30.19	13.03	13.34	6.52	6.09	2.30	4.31	0.90	1.40
117.51	61.98	47.44	8.91	6.54	2.30	4.41	10.41	21.67
18.13	6.80	6.76	1.35	4.48	2.30	5.70	0.10	9.69
156.42	74.68	65.16	30.13	28.23	2.30	32.07	48.79	66.92
10.11	4.42	4.55	2.60	1.68	2.33	2.68	3.38	8.35
24.38	10.69	12.74	4.85	2.95	2.34	50.30	44.71	16.91
10.94	4.60	5.60	0.95	2.98	2.34	1.46	2.75	5.81
900.24	437.99	374.63	173.66	147.11	2.34	1.79	3.37	5.49
34.76	14.76	14.91	5.78	7.93	2.34	1.47	3.41	8.87
18.23	9.02	9.20	5.78	1.87	2.35	7.46	0.10	12.88
22.97	8.73	12.56	17.83	4.64	2.36	3.26	1.60	6.53
60.31	27.17	22.48	10.32	10.18	2.36	2.85	2.42	8.15
80.02	35.70	31.62	13.53	16.72	2.36	0.76	0.10	1.08
3.95	2.42	1.78	0.80	0.10	2.37	4.13	4.76	12.71
8.65	2.84	3.61	2.98	1.04	2.37	2.64	0.10	5.12
19.35	8.75	8.34	7.64	0.10	2.38	6.39	5.67	22.60
7.26	3.32	2.61	0.76	1.69	2.39	1.87	1.04	5.43
44.61	18.96	19.76	9.79	8.57	2.40	0.27	3.09	0.27
25.85	9.33	13.63	1.92	2.32	2.41	3.90	1.92	9.71
794.57	284.37	379.54	106.44	115.71	2.42	2.37	0.30	3.60
33.52	11.64	15.02	5.03	3.23	2.42	0.27	1.33	2.25
100.58	48.06	35.90	14.29	21.29	2.44	1.22	1.69	2.54
47.38	17.55	18.80	9.11	6.49	2.47	1.45	0.12	2.00
90.64	46.05	28.36	7.55	7.54	2.48	3.96	3.92	12.19

39.11	10.84	16.24	21.41	8.13	2.48	9.80	6.63	22.13
8.88	3.50	2.61	1.74	0.41	2.50	1.44	8.20	2.98
19.13	10.16	6.50	4.52	1.09	2.51	5.59	10.80	30.91
32.04	14.32	9.94	3.86	5.84	2.53	4.63	2.83	11.40
0.46	0.53	0.38	7.25	1.89	2.54	4.20	1.80	3.77
41.67	17.18	13.75	4.05	4.44	2.55	2.58	1.87	8.42
14.93	5.94	6.13	3.01	5.10	2.56	1.67	1.24	0.38
7.79	3.54	5.47	44.73	1.75	2.57	6.59	6.67	26.78
18.83	9.44	5.21	1.86	2.27	2.58	0.82	1.63	1.88
34.09	10.76	16.52	5.20	3.58	2.58	3.03	5.52	11.22
646.95	195.91	284.79	63.80	69.16	2.62	0.93	3.48	5.89
13.72	4.65	4.77	1.53	1.88	2.63	6.59	2.34	9.73
56.84	18.03	20.25	9.61	4.63	2.63	2.06	1.04	4.64
30.91	6.79	10.21	2.82	2.63	2.69	6.47	5.71	21.65
53.30	16.90	19.55	7.83	9.07	2.69	1.32	2.95	4.27
30.86	11.22	11.81	1.11	0.55	2.72	5.46	2.07	0.52
62.38	22.37	25.15	7.01	10.40	2.74	1.50	1.99	5.63
24.20	8.25	8.39	3.12	5.07	2.74	2.35	3.12	13.38
667.12	321.05	163.05	66.62	85.40	2.74	4.27	3.82	0.61
0.84	0.44	0.70	0.45	0.57	2.75	3.06	4.59	14.10
23.12	12.18	6.60	3.41	3.06	2.76	1.47	1.11	6.00
8.73	3.20	4.07	1.16	1.22	2.78	4.65	4.09	24.63
13.01	8.02	5.22	4.80	0.89	2.82	0.73	1.11	0.92
21.16	35.53	26.47	6.89	7.49	2.84	0.95	5.51	0.39
27.94	14.92	7.05	3.55	3.51	2.84	1.49	1.97	2.55
29.11	14.29	5.98	2.37	1.14	2.86	1.72	0.27	0.45
439.10	183.68	101.23	43.06	46.39	2.88	4.10	0.51	12.50
30.62	6.68	15.54	1.45	2.53	2.89	2.40	3.82	0.72
63.81	30.51	13.99	5.77	6.15	2.89	5.35	0.90	0.24
48.13	15.41	16.83	4.44	7.41	2.91	0.90	1.13	0.74
60.75	28.77	14.97	2.72	2.87	2.98	0.37	4.47	0.83
30.12	13.58	10.07	5.91	3.66	3.01	1.20	3.89	0.67
148.43	12.83	36.56	1.19	0.85	3.02	2.38	2.21	2.65
33.52	8.91	12.45	5.03	3.23	3.02	1.26	1.76	11.10
5.04	1.22	1.40	2.59	1.39	3.03	2.44	2.68	9.56
27.44	7.97	9.13	3.93	2.00	3.06	1.36	4.02	3.80
0.80	0.37	0.20	5.87	0.10	3.08	2.03	0.89	4.52
45.42	10.45	17.79	1.89	3.55	3.09	0.53	0.44	0.76
28.32	8.42	6.34	1.77	2.33	3.09	3.82	10.06	1.01
7.53	7.49	11.41	0.83	1.90	3.13	0.43	2.26	11.23
10.38	1.06	3.86	0.64	0.47	3.14	0.40	3.49	1.25
91.97	23.66	26.97	10.46	11.36	3.15	0.34	6.67	1.26
43.14	15.65	18.28	2.97	9.47	3.15	6.75	2.18	0.58
32.27	9.91	10.84	1.79	3.24	3.17	0.86	7.77	13.95
26.25	4.88	9.33	3.13	5.10	3.18	1.08	0.86	1.11
6.19	0.98	2.25	0.55	0.43	3.21	3.47	8.52	68.48
408.85	2016.97	332.42	471.96	500.82	3.22	3.08	1.63	0.25
2.17	0.94	0.25	1.37	2.84	3.22	2.79	2.60	10.97
195.58	52.31	57.35	26.98	21.12	3.26	0.69	2.38	1.87
6.55	2.59	2.01	1.88	0.33	3.26	0.88	6.87	0.51
19.83	6.09	7.96	1.04	2.14	3.29	3.29	1.22	1.82
2.50	0.30	1.40	3.60	1.02	3.30	3.71	0.32	2.29

4.43	0.31	1.51	2.14	0.48	3.33	3.12	2.59	32.34
248.19	135.94	141.36	18.49	5.81	3.34	0.55	0.47	1.59
358.03	135.29	77.95	19.52	18.29	3.34	1.62	3.68	2.04
46.72	18.85	6.01	0.75	0.98	3.35	6.84	0.52	0.89
13.40	2.99	4.60	1.51	1.10	3.35	0.45	1.18	2.10
11.35	3.09	2.24	1.77	3.97	3.35	7.76	8.21	1.24
5.91	1.65	1.23	0.80	2.52	3.46	1.67	1.92	38.43
12.71	3.55	3.81	0.58	1.58	3.49	1.96	0.97	2.22
22.99	7.11	4.31	4.21	3.67	3.51	3.78	5.46	84.01
15.62	6.01	3.89	1.08	2.10	3.61	0.65	1.45	15.24
5.46	2.65	2.32	1.37	1.16	3.63	1.96	1.25	0.20
3.65	1.08	0.91	0.92	1.15	3.74	7.24	6.21	1.52
1.34	5.00	2.20	1.58	4.46	3.74	0.97	0.29	1.92
2.02	1.08	0.20	1.72	1.78	3.83	0.52	0.64	2.29
40.98	8.33	9.13	4.07	2.85	3.84	0.95	3.47	2.10
156.03	50.38	33.65	1.48	0.95	3.91	2.84	1.19	21.44
5.35	0.34	1.45	1.10	0.43	4.16	1.42	4.21	3.84
2.29	0.29	0.94	5.35	1.85	4.22	0.65	4.72	2.35
20.04	13.25	2.19	1.17	2.40	4.23	1.26	1.73	16.47
3.94	0.28	1.13	1.14	0.90	4.26	2.68	11.77	2.86
3.53	0.83	0.45	0.94	0.74	5.44	0.39	1.06	3.28
319.36	63.17	45.84	3.47	7.71	5.60	34.31	1.90	2.49
71.26	119.02	204.73	214.11	260.62	5.75	0.64	4.83	2.80
2.82	0.20	0.70	0.86	1.37	6.08	0.25	0.47	3.38
4.96	0.43	0.91	1.31	0.48	6.10	9.42	0.55	1.66
36.08	5.54	4.68	1.21	0.98	6.95	0.90	2.71	3.96
5.28	1.11	0.56	2.11	2.61	7.58	77.69	0.75	6.14
126.12	17.74	16.68	8.76	1.29	7.63	2.57	1.10	6.89
4.83	0.78	0.46	0.05	0.77	10.85	6.04	2.63	10.03
5.99	0.20	0.71	4.18	3.64	11.85	0.91	3.76	18.70

CBZ_2
4.53
8.89
0.75
0.31
0.97
0.62
1.60
1.54
3.69
0.66
0.68
3.18
0.56
2.79
1.47
0.39
0.47
1.74
0.26
1.63
6.03
2.19
0.43
21.97
0.78
1.35
1.69
0.88
1.04
1.78
0.97
0.40
3.92
1.89
6.32
0.40
28.37
1.12
6.17
1.06
6.17
5.42
2.08
3.72
12.30
6.36
6.39
3.10
4.21
10.99

5.07
3.82
3.78
3.70
3.56
0.24
69.90
1.66
25.37
3.45
15.48
8.54
3.24
10.32
22.57
13.82
18.88
2.21
1.01
14.26
19.81
9.92
39.92
19.11
0.46
22.98
37.22
31.06
2.85
2.59
9.31
7.69
8.19
11.22
25.62
1.07
10.50
4.46
8.19
7.23
8.21
3.13
2.17
6.64
2.72
6.94
1.59
11.34
14.06
17.85
5.68
3.52

4.75
7.15
4.49
2.98
6.98
12.41
38.41
34.69
9.27
6.72
12.51
3.65
8.80
4.40
36.00
11.36
19.07
14.38
44.00
4.55
8.92
1.80
20.37
19.00
25.32
16.55
46.48
2.76
11.47
16.16
18.65
14.97
7.47
5.90
7.82
35.60
11.25
16.36
19.46
19.53
7.93
4.09
11.00
19.64
8.97
2.67
3.43
8.82
18.25
13.01
32.19
34.75

43.57
19.15
4.13
2.33
6.52
10.34
9.64
60.74
18.98
13.47
11.84
7.71
11.07
7.94
4.20
22.27
5.54
6.94
16.86
12.99
33.52
30.62
2.65
4.51
19.04
8.80
9.45
11.97
1.33
18.87
6.37
53.39
8.70
22.16
6.07
9.12
5.09
8.85
7.58
7.24
1.46
13.56
3.01
13.38
3.26
0.34
8.00
4.06
2.77
3.09
2.91
12.91

31.15
2.06
24.14
13.90
2.69
6.79
0.31
20.02
1.37
19.21
9.85
22.51
6.69
23.06
7.27
0.26
8.38
8.71
0.21
19.08
5.34
17.04
3.11
0.58
7.55
0.55
8.35
0.28
0.80
0.37
0.29
0.45
1.62
7.64
22.23
5.55
9.54
0.61
0.42
5.95
0.31
0.34
1.05
10.03
0.62
35.25
1.61
45.51
0.24
1.76
2.32
0.33

42.32
1.06
0.69
1.84
0.68
35.67
16.19
1.01
67.99
19.50
3.15
2.06
1.73
1.41
1.67
4.29
0.70
2.00
13.66
2.33
2.15
3.04
2.75
2.47
4.79
4.44
4.03
5.22
4.67
40.48
